# Thermally Activated Delayed Fluorescence: Polarity, Rigidity, and Disorder in Condensed Phases

**DOI:** 10.1021/jacs.2c05537

**Published:** 2022-08-09

**Authors:** D. K. Andrea Phan Huu, Sangeeth Saseendran, Rama Dhali, Larissa Gomes Franca, Kleitos Stavrou, Andrew Monkman, Anna Painelli

**Affiliations:** † Department of Chemistry, Life Sciences and Environmental Sustainability, University of Parma, 43124 Parma, Italy; ‡ Department of Physics, Durham University, South Road, Durham DH1 3LE, U.K.

## Abstract

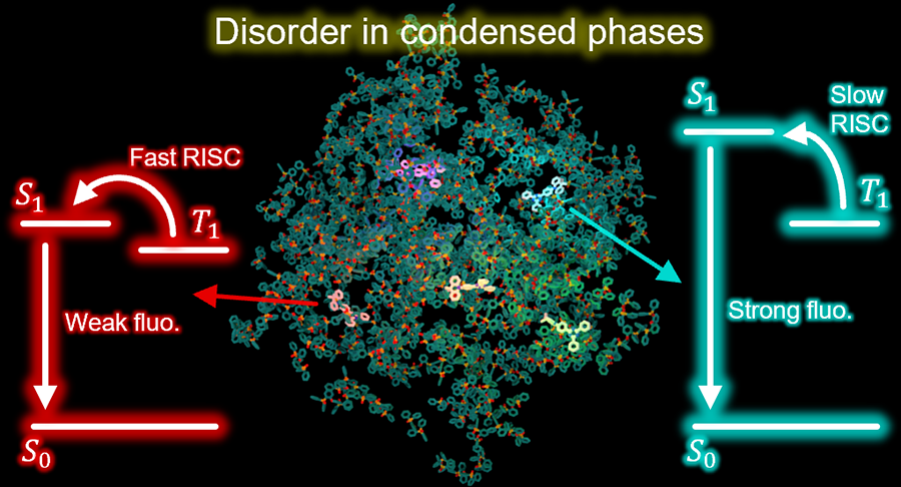

We present a detailed and comprehensive picture of the photophysics of thermally activated delayed fluorescence (TADF). The approach relies on a few-state model, parametrized *ab initio* on a prototypical TADF dye, that explicitly accounts for the nonadiabatic coupling between electrons and vibrational and conformational motion, crucial to properly address (reverse) intersystem crossing rates. The Onsager model is exploited to account for the medium polarity and polarizability, with careful consideration of the different time scales of relevant degrees of freedom. TADF photophysics is then quantitatively addressed in a coherent and exhaustive approach that accurately reproduces the complex temporal evolution of emission spectra in liquid solvents as well as in solid organic matrices. The different rigidity of the two environments is responsible for the appearance in matrices of important inhomogeneous broadening phenomena that are ascribed to the intertwined contribution from (quasi)static conformational and dielectric disorder.

## Introduction

Thermally activated delayed fluorescence (TADF) is an old phenomenon^1^ that recently acquired wide popularity thanks to the seminal work of Adachi et al.,^2,3^ who proposed TADF as a way to harvest triplet states in organic light-emitting diodes (OLED), bringing their theoretical internal efficiency from a disappointing 25% to 100%. In OLED the dark triplet excitons are produced with an efficiency 3 times larger than the bright singlet excitons, but in TADF systems, the very small energy gap between singlet and triplet states allows for a reverse intersystem crossing (RISC) phenomenon, populating the bright singlets. As a spin-forbidden process, RISC is slow, and TADF shows itself with a delayed fluorescence, typically in the microsecond time scale. TADF is nowadays popular in the OLED community,^4−21^ but it also finds interesting applications in bioimaging where long fluorescence lifetimes allow for the observation of long-lived phenomena.^22,23^

Donor–acceptor (DA) molecules, where an electron donor moiety is linked to an acceptor moiety via a poorly conjugated bridge, are among the most studied structures for TADF applications.^3^ The simplest model to describe DA pairs dates back to 1958, when Mulliken proposed a two-state model to describe charge-transfer (CT) absorption in halogen–benzene complexes.^24^ In the singlet subspace, two diabatic states describe the low-energy physics of a DA pair, a neutral DA, and a charge-separated (zwitterionic) D^+^A^–^ state. The charge resonance (conjugation) between the two fragments mixes the two states and is responsible for the widening of the optical gap and for the finite oscillator strength of the CT state. The zwitterionic triplet state stays unmixed. Accordingly, in poorly conjugated DA pairs, where the lowest singlet state (S_1_) is an almost pure zwitterionic state, a small singlet–triplet (ST) gap is expected. However, poorly conjugated systems are poorly fluorescent due to the small oscillator strength of S_1_ and are characterized by a negligibly small S_1_–T_1_ spin–orbit coupling (SOC).^25^ Efficient TADF requires at the same time a small ST gap, large SOC matrix elements, and a large oscillator strength. The tight competition among these stringent requirements suggests that obtaining efficient TADF is a very delicate task.

The Mulliken picture, while inspiring, is far too simple to address TADF. Vibrational and conformational motions play a pivotal role in favoring ISC and RISC processes.^26−29^ Moreover, other electronic states, typically triplet states localized on either the D or A unit, may be not too far in energy from the triplet CT state, leading to triplet states with mixed character.^7,26,30^ The emerging picture, already fairly complex, is made even more problematic by environmental effects.^9,17,31^ TADF is often experimentally investigated in solution, where the polarity and polarizability of the solvent nontrivially affect the dye photophysics. More dramatically, in OLED the dyes are embedded in solid matrices whose polarity, polarizability, rigidity, and disorder all enter into play.^10,32−38^

**Figure 1:**
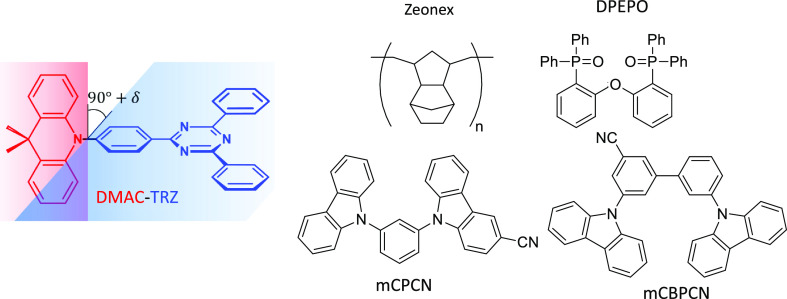
Kekulé structures of DMAC-TRZ (left) and hosts used in this work (right).

Here we build on previous work^31,39^ on a prototypical DA dye for TADF application, 9,9-dimethyl-9,10-dihydroacridin-4,6-triphenyl-1,3,5-triazine (DMAC-TRZ, Figure 1), to discuss environmental effects. Specifically, we adopt the essential state model proposed and parametrized *ab initio* for DMAC-TRZ.^31^ The model is exploited to calculate RISC and ISC rates following an original strategy that fully accounts for the anharmonic and nonadiabatic vibrational and conformational motion.^39^ This sets a sound basis to address environmental effects on TADF, as needed to directly compare with experimental data. The model accurately describes in a single unifying theoretical framework steady-state and time-resolved spectral properties of DMAC-TRZ when dissolved in liquid solvents and in solid matrices with different polarity. The intriguing and highly nontrivial time dependence of emission spectra collected in several matrices is quantitatively reproduced, accounting for the subtle interplay among polar solvation dynamics, the nonadiabatic dynamics of conformational degrees of freedom fully accounting for static dielectric and conformational disorder, and the consequent inhomogeneous broadening phenomena. The model, quantitatively validated against experimental photoluminescence data collected in diluted solutions and solid matrices, sets a solid basis to account for environmental effects, including disorder, in phenomenological models for OLED devices.^40,41^

## Materials and Methods

### Molecular Model

**Figure 2:**
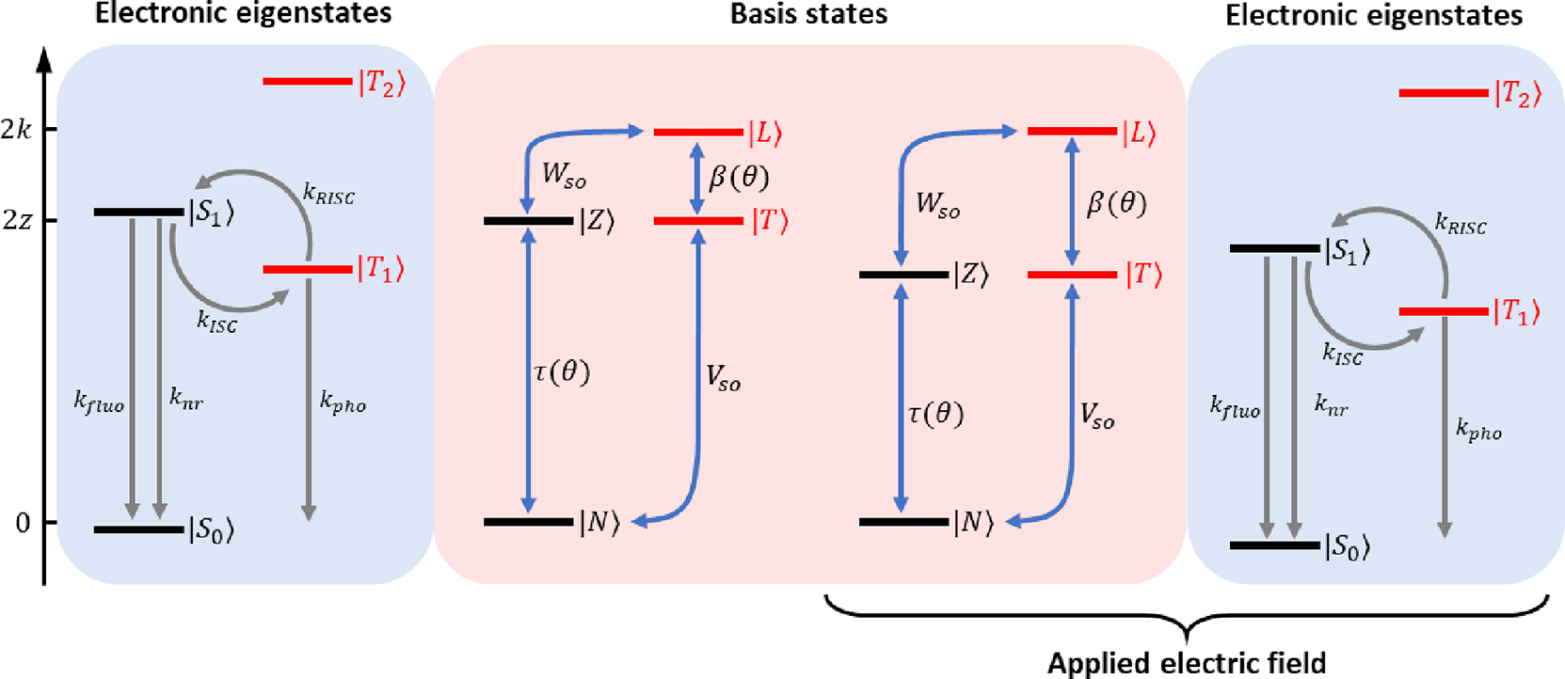
DMAC-TRZ model. The two middle panels in the pink shadowed area show the four basis states in the absence and in the presence of an applied field, the field lowers (by the same amount) the energy of the Z and T states. The two lateral panels (blue shadowed areas) show the energies of the eigenstates calculated in the absence and in the presence of the field (left and right blue-shadowed areas, respectively). The field lowers the energy of T state, leading to decrease of the T–L coupling, and hence to a reduction of the ST gap.

As detailed in ref (31) and shortly introduced above, we consider only two electronic basis (diabatic) singlet states, corresponding to the neutral DA state, |N⟩, and to the zwitterionic D^+^A^–^ state, |Z⟩. As sketched in Figure 2, an energy gap 2*z* separates the neutral and the zwitterionic states. The two singlet states are mixed by a matrix element τ to give the ground state S_0_ and the first excited singlet S_1_. In DMAC-TRZ the amount of mixing is small, so that S_0_ closely resembles |N⟩ and S_1_ closely resembles |Z⟩. Accordingly, S_1_ is also often termed a CT state. In the triplet subspace we account for two basis states: the zwitterionic state, |T⟩, at the same energy as |Z⟩ and an effective local state, |L⟩, with 2*k* measuring the energy difference between the two basis triplet states. The two triplet states are mixed by a matrix element β, so that the two lowest excited triplets T_1_ and T_2_ are mixtures of the CT and LE triplet. The singlet and triplet subspaces can be treated separately because SOC matrix elements are very small and marginally affect the nature of the states. However, they are of paramount importance to model TADF. Then, according to the El-Sayed rule, we introduce *V*_SO_, the SOC between |N⟩ and |T⟩ and *W*_SO_ the SOC between |Z⟩ and |L⟩.

Vibrational coupling must be introduced to reproduce spectral band shapes. To this effect, we introduce an effective molecular vibration with frequency ω_*v*_ in the mid-infrared region, which modulates the energy of the zwitterionic states as , where *Q̂* = *â*^†^ + *â* is the dimensionless coordinate and ϵ_*v*_ is the energy gained by the system upon the geometrical rearrangement of the molecule when going from the |N⟩ state to either the |Z⟩ or |T⟩ states.

Conformational motion is very important in DMAC-TRZ: at the ground-state equilibrium, the planes spanned by the DMAC and TRZ units are mutually orthogonal, but the rotation of the dihedral angle δ̂ = *d̂*^†^ + *d̂* around the orthogonal equilibrium position (δ = 0) is very important to drive ISC and RISC. A quartic potential is introduced to describe this motion, with harmonic frequency ω_*c*_ and quartic coefficient *a*. Orthogonal D and A are not conjugated, but the conjugation increases when moving away from orthogonality. This is accounted for introducing a δ dependence of the mixing matrix elements: τ(δ) = τ_0_|sin(δ)| and β(δ) = β_0_|sin(δ)|. With these definition, the total molecular Hamiltonian reads^31^
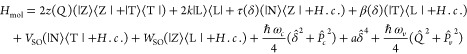
1where *P*_*v*_ and *P*_*c*_ are the conjugated momenta with the vibrational and conformational coordinate, respectively. A detailed discussion of the model and its parametrization against TD-DFT results can be found in ref (31) (see also the Supporting Information). Model parameters are listed in Table 1.

**Table 1:** Model Parameters (eV) from Ref (31)

*z*	τ_0_	*k*	β_0_	*V*_soc_	*W*_soc_	ω_*c*_	*a*	ω_*v*_	ϵ_*v*_
1.72	0.75	1.96	0.85	3.84 × 10^–4^	1.74 × 10^–4^	2.40 × 10^–4^	1.43 × 10^–7^	0.18	0.17

To calculate optical spectra and to address environmental effects, we need a definition of the dipole moment operator. We use the Mulliken definition of the dipole moment as a purely electronic operator, whose only nonvanishing matrix element on the diabatic basis is μ_0_, the dipole moment associated with the zwitterionic states.^24,42^ From a detailed analysis of TD-DFT results (and specifically from the slope of the CT transition frequency versus an applied electric field) we estimate μ_0_ ∼ 27.02 D.^31^

### TADF in a Dielectric Medium

A dye inserted in a dielectric medium (a solvent or a matrix) is affected by the electric fields generated by the medium in response to the presence of the dye. Solvatochromism, that is, the dependence of the spectral properties of dyes on the solvent, is the most obvious manifestation of the phenomenon.^43^ Models accounting for normal and inverse solvatochromism in polar dyes have been known for decades,^43,44^ and more detailed treatments also accounting for the evolution of spectral band shapes have been proposed.^42,45,46^ In TADF dyes, the phenomena are more complex because not just the spectra depend on the dielectric properties of the medium,^31,39^ but the ST gap and the SOC are also affected with important and highly nontrivial consequences on the TADF photophysics.^9,10,17^ To make the issue more complex, delayed fluorescence occurs on very long time scales that in solid matrices are possibly comparable to the matrix relaxation times, resulting in a highly nontrivial interplay of interactions.

To describe the interaction between the dye and its environment, we adopt the same model traditionally and successfully adopted to address solvatochromism; that is, we describe the dye as a point dipole embedded in a continuum dielectric medium.^42,44,47^ In this approximation, when dispersed in a dielectric medium, the dye experiences an electric field, called the reaction field, proportional to the dipole moment of the dye. Of course, the proportionality constant depends on the dielectric properties of the medium. As long as we are interested in spectral properties in the visible region, the response of the medium is conveniently partitioned in two contributions with different dynamics.^42,44,47^

Specifically, a fast component of the reaction field is related to the solvent polarizability, that is, to the rearrangement of the electronic clouds of the medium molecules in the close proximity of the dye and is governed by the solvent refractive index η (the square root of the dielectric constant at optical frequencies). To build an effective solvation model, fast solvation will be dealt with in the antiadiabatic approximation, that is, assuming that the solvent electronic clouds readjust instantaneously to the charge fluctuations in the dye (at least in the spectral region of interest). This approximation holds as long as the electronic excitations of the solvent are well separated in energy from the relevant solute excitations,^48−50^ a good approximation for most solvents, which can become delicate for some matrices characterized by low-lying absorptions. In this approximation, the solvent polarizability is accounted for by a renormalization of the model Hamiltonian and specifically of the *z* parameter that becomes dependent on the medium refractive index (see the Supporting Information).^39,49^ In organic media the refractive index spans a narrow range leading to a narrow variability of *z* (see the Supporting Information). In DMAC-TRZ a sizable reduction of RISC and ISC rates is calculated when going from the gas phase to a medium,^39^ pointing to the need to explicitly account for environmental effects when comparing experimental and theoretical results.

The second component of the reaction field is associated with the vibrational and orientational motions of polar molecules in the medium. Relevant degrees of freedom are slow and can be treated in the adiabatic approximation, that is, neglecting the kinetic energy associated with *F*_*or*_. The Hamiltonian that describes the dye in a dielectric environment then reads
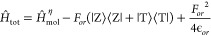
2where *Ĥ*_mol_^η^ is the molecular Hamiltonian in eq 1, but with renormalized *z* as to account for the medium refractive index, and *F*_*or*_ is the reaction field component related to the slow degrees of freedom of the medium. The second term on the right-hand side of eq 2 describes the lowering of the energy of the zwitterionic states in the presence of the field (see Figure 2). To keep the equations simple, the electric field is actually measured in energy units, by multiplying it by μ_0_, the dipole moment of the zwitterionic states. Finally, the last term is the elastic energy associated with the reaction field, where the restoring force goes with the solvent relaxation energy, ϵ_*or*_, a parameter that accounts for the medium polarity (cf. the Supporting Information).

The above equations, derived in ref (42), were extensively adopted to describe linear and nonlinear spectral properties of polar dyes in different environments. Here we underline that the relation between the restoring force for *F*_*or*_ and ϵ_*or*_ is fixed by imposing that in each state the equilibrium *F*_*or*_ is proportional to the molecular dipole moment (in turn, proportional to the weight of the zwitterionic |T⟩ and |Z⟩) states:
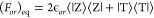
3In DMAC-TRZ the S_0_ state is largely neutral, so that at the equilibrium *F*_*or*_ ∼ 0 in all solvents or matrices. In S_1_ instead the molecular dipole moment is large, and a sizable equilibrium *F*_*or*_ value is expected, which increases in more polar environments (at least as long as the environment can relax after the photoexcitation of the dye). There is, however, another important effect of polar solvation. At finite temperature, not just the *F*_*or*_-equilibrated state is populated, but a distribution of *F*_*or*_ is expected that can be calculated in terms of a Boltzmann distribution of *F*_*or*_-dependent energies. This distribution is responsible for inhomogeneous broadening phenomena in polar solvents.^42,45^ Indeed, in nonpolar or weakly polar solvents ϵ_*or*_ is close to zero, so that the restoring force for *F*_*or*_ is very large, leading to narrow distributions around the equilibrium: inhomogeneous broadening due to polar solvation is marginal in these conditions, explaining the partially resolved structure of CT absorption and fluorescence bands of polar dyes in nonpolar environments.^45^ As the solvent polarity increases, the *F*_*or*_-restoring force decreases and the resulting broader *F*_*or*_ distributions are responsible for the gradual broadening of CT absorption and emission bands with increasing the polarity of the environment.^45^ In the specific case of DMAC-TRZ with a largely neutral ground state, the distribution is always centered around *F*_or_ ∼ 0, but it becomes broader and broader with increasing ϵ_*or*_, spanning regions with positive and negative *F*_*or*_ (see Figure S1). S_1_ and T_1_ states have similar polarity and similar distributions centered at *F*_*or*_ values that increase with ϵ_*or*_.

## Results and Discussion

### TADF in Liquid Solvents

The numerically exact, nonadiabatic solution of the molecular Hamiltonian *H*_mol_^η^ in eq 2 is obtained diagonalizing the Hamiltonian matrix written on the basis direct product of the four diabatic states times the eigenstates of the harmonic oscillators associated with the vibrational and conformation motions.^39^ Of course, the oscillator basis must be large enough to reach convergence. As for the vibrational mode, 10–20 basis states are enough, but the very low frequency of the conformational mode requires 600 or more states, for an overall dimension exceeding 20,000 states.

Once the Hamiltonian is diagonalized, we calculate the transition frequencies and dipole moments among the vibronic states (the Hamiltonian eigenstates) to address optical spectra. Specifically, to calculate absorption spectra, the lowest vibronic states of the ground-state manifold are populated accounting for the Boltzmann distribution. Accordingly, we obtain an *F*_*or*_-dependent energy, and *F*_*or*_-dependent absorption spectra are calculated by averaging over the spectra calculated starting from each vibronic state. Finally, the global absorption spectrum is obtained by averaging *F*_*or*_-dependent spectra accounting for the Boltzmann distribution relevant to the *F*_*or*_-dependent energy.

To address fluorescence, we recognize that in liquid solvents the dynamics associated with slow solvation typically occur in the picosecond time window, not experimentally accessible in this study. In any case, solvent relaxation is much faster than fluorescence lifetimes (typically in the nanosecond regime), and steady-state fluorescence is dominated by the signal from the excited dye surrounded by the equilibrated solvent. Therefore, the calculation of fluorescence spectra goes along the same lines as described for absorption spectra but accounting for each *F*_*or*_ of the emission from each vibronic state in the excited singlet subspace and averaging over the relevant *F*_*or*_ distribution.

**Figure 3:**
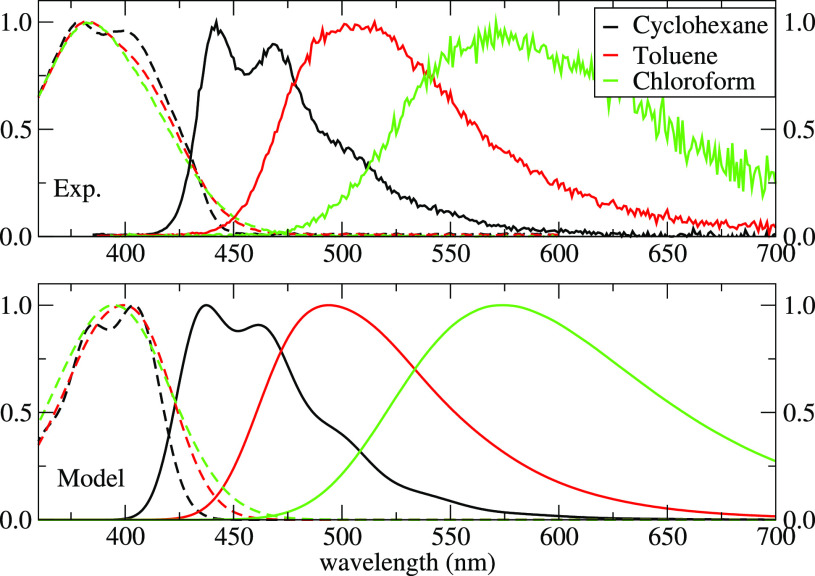
Normalized absorption and fluorescence spectra (dashed and continuous lines, respectively) of DMAC-TRZ in cyclohexane, toluene, and chloroform. Top panel: experimental data. Bottom panel: calculated spectra. The ϵ_*or*_ values are adjusted to best reproduce the experimental emission spectra: ϵ_*or*_ (cyclohexane) = 0.10 eV, ϵ_*or*_ (toluene) = 0.22 eV, and ϵ_*or*_ (chloroform) = 0.40 eV. The HWHM associated with each vibronic transition is Γ = 0.03 eV.

Figure 3 shows calculated spectra for DMAC-TRZ in different solvents. The molecular parameters are listed in Table 1.^31^Table 2 lists, for the three solvents, the values of ϵ_*el*_, obtained from eq 1, and of ϵ_*or*_, adjusted to best reproduce experimental spectra.^31^ While not perfect, the agreement with the experiment is fairly good, in view of the simplicity of the adopted model. Specifically, calculated absorption spectra do not account for high-frequency absorption bands that are partly superimposed to the low-frequency band and may alter its shape. We underline that absorption spectra calculate treating the conformational mode in the adiabatic approximation (see the Supporting Information) coincide with the spectra in Figure 3. Indeed, even if the nonadiabatic calculation does not rely on a static δ distribution, it implicitly accounts for the disorder on the (dynamical) δ variable (see Figure S2).

**Table 2:** Solvent Parameters (from Ref (31)) and Estimated *k*_RISC_ and *k*_ISC_

	cyclohexane	toluene	chloroform
ϵ_*el*_ (eV)	0.246	0.242	0.253
ϵ_*or*_ (eV)	0.10	0.22	0.40
*k*_RISC_ (s^–1^)	4.87 × 10^4^	3.10 × 10^5^	2.19 × 10^6^
*k*_RISC_^exp^ (s^–1^)	6.05 × 10^4^	2.71 × 10^5^	2.69 × 10^6^
*k*_ISC_ (s^–1^)	4.18 × 10^5^	1.78 × 10^6^	1.11 × 10^7^
*k*_ISC_^exp^ (s^–1^)	1.33 × 10^7^	2.63 × 10^7^	1.77 × 10^7^

**Figure 4:**
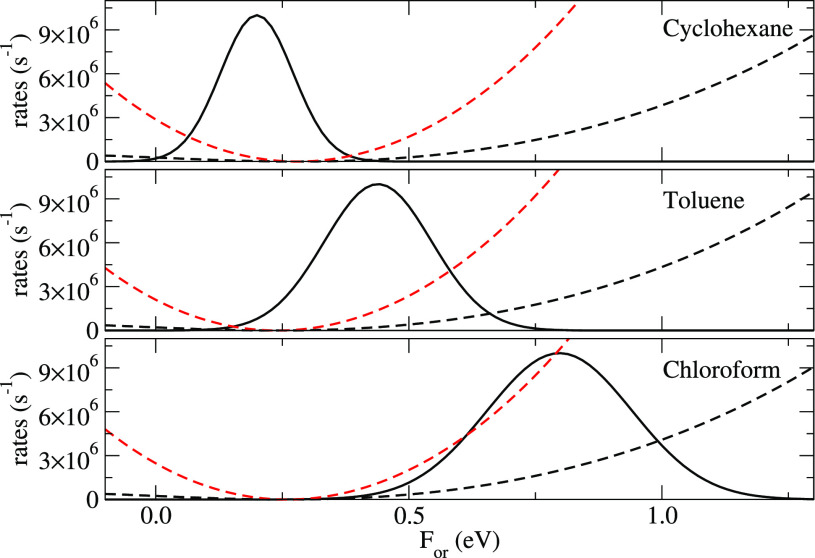
Normalized triplet distribution of DMAC-TRZ vs *F*_*or*_ (continuous lines) in cyclohexane, toluene, and chloroform, superimposed to *k*_ISC_ and *k*_RISC_ (red and black dashed lines, respectively).

The adiabatic treatment of δ, which works well for absorption and fluorescence spectra, fails when applied to RISC and ISC calculations because the ST gap is comparable to conformational energies. We then proceed diagonalizing, for each *F*_*or*_ value, the complete nonadiabatic Hamiltonian and then calculate rates among each pair of vibronic states using the state-by-state Fermi golden rule.^39^ The overall rate is finally obtained as the thermal average over the initial state population (the lowest triplet state for RISC and the lowest excited singlet state for ISC). Again the calculations are repeated for different values of *F*_*or*_ to obtain *F*_*or*_-dependent RISC and ISC rates; the relevant results are shown as red and black dashed lines in the three panels of Figure 4.

In liquid solvents, the orientational relaxation times are faster than all photophysical processes of interest. Accordingly, the solvent is always equilibrated with the solute, and the overall rate for the generic process from state *i* to state *f* can be calculated as the thermal average over the *F*_*or*_ distribution equilibrated to the *i* state.^51^ The overall *k*_RISC_ rate in each solvent is then calculated by summing over the *F*_*or*_-dependent RISC rates, weighted by the *F*_*or*_ distribution equilibrated to T_1_. The same calculation is done for the ISC rates, accounting for the *F*_*or*_ distribution relevant to S_1_ state. Continuous lines in Figure 4 show the *F*_*or*_ distribution relevant to T_1_ (the S_1_ distribution being marginally different). Upon increasing the solvent polarity, the distribution moves toward *F*_*or*_ values where both RISC and ISC rates increase, explaining the overall increase of both rates upon increasing the solvent polarity.

Table 2 compares calculated RISC and ISC rates with experimental RISC, ISC, and fluorescence rates obtained from the biexponential fit of the integrated luminescence intensity measured from diluted (0.8 mM) degassed solutions of DMAC-TRZ in methylcyclohexane, toluene, and chloroform (Figure S5). RISC rates are well in line with experimental results, showing a progressive increase with the solvent polarity. As for ISC, we somewhat underestimate the rates in nonpolar solvents and indeed predict a steady increase of ISC rates with the solvent polarity, while experimental results point to a marginal dependence of ISC rates on the solvent polarity.

Because in solution all molecular and solvent relaxation processes are much faster than all photophysical processes, the emission spectra do not show any appreciable time dependence (Figure S4), and the luminescence intensity decay follows a well-behaved biexponential curve, the tail observed at long delays in toluene being ascribed to triplet-triplet annihilation (Figure S5).

### TADF in Organic Matrices

#### Conformational and Polar Disorder

The photophysics of TADF dyes dispersed in organic matrices (see Figure 1 for representative structures) is far more complex than in liquid solutions. Fairly extensive studies are available for DMAC-TRZ in several matrices (cf. Figure S7).^34^ Time-resolved emission spectra of DMAC-TRZ in organic amorphous matrices measured at room temperature show a red-shift in the first 80–90 ns (prompt fluorescence regime). The magnitude of this initial red-shift increases with the host polarity. Moreover, the emission spectrum measured at the first accessible time, *t* ∼ 2.3 ns, moves to the red with the host polarity, suggesting a partial host rearrangement in a time scale not experimentally accessible. During delayed fluorescence, the emission band strongly blue-shifts in DPEPO, weakly blue-shifts in Zeonex and mCPCN, and does not move in mCBPCN (cf. Figure S7). Moreover, in all matrices a clear nonexponential tail is observed in the time evolution of the emission intensity. This complex behavior points to inhomogeneous broadening effects as well as to a complex interplay between the concurrent dynamics of processes that include fluorescence, phosphorescence, nonradiative decay, RISC, ISC, the conformational motion, and the dynamics of the matrix itself. Quite interestingly, nonexponential decays are typically observed in dynamical processes where different species concur to the observed fluorescence or when a slow dynamical process affects the system dynamics.^52−54^

There are two main sources of quasi-static disorder and for dyes in matrices. In the first place, the conformational motion of the dye is hindered in rigid matrices, and a static distribution of the dihedral angle must be accounted for rather than a dynamic distribution, as considered in liquid solutions. Moreover, in polar matrices, static disorder in the local electric field generated by the configuration of polar groups in the matrix molecules around the dye is an important source of inhomogeneous broadening. In this subsection we will address the two sources of disorder separately, just to understand their role. In the next subsection, we will describe the complete system in an effort to address experimental data.

Organic matrices are rigid structures that do not allow for the full conformational relaxation of the dye upon relaxation.^55,56^ Moreover, the dye entrapped in the matrix may be frozen in nonequilibrium geometries. To account for the reduced conformational freedom of the dye inside the matrix, we modify the conformational potential in eq 1. Specifically, we set *a* = 0 to restore the harmonic approximation, a good approximation for small-amplitude motions, and consider a stiff potential with a larger ω_*c*_ than in solution. To account for conformational disorder, for each dye in the matrix we assume small oscillations of the dihedral angle around a different equilibrium positions, so that the conformational potential reads
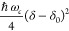
4We will assume a Gaussian distribution of δ_0_, in line with results from molecular dynamics calculations,^57^ and to account for the matrix rigidity, we will maintain the distribution frozen upon excitation. In the following, results are reported for ω_*c*_ = 4.0 × 10^–3^ eV, which corresponds to average δ oscillations of about ±3° at ambient temperature. Rates computed for different values of ω_*c*_ are shown in the Supporting Information. Varying ω_*c*_ leads to small variations of ISC and RISC rates (Figure S9) that will marginally affect the overall simulation in the next subsection.

Dielectric disorder is more subtle. In liquid solvents the major contributions to polar solvation arises from the tumbling of the polar molecules around the solute. This orientational motion is very fast in liquid solvents (picosecond time scale), but it becomes slow and possibly totally hindered inside the solid matrices of interest. However, partial rearrangements (torsion of small groups or lateral chains, vibrational relaxation) can still occur in matrices in the time scale of interest for the TADF photophysics. Indeed, in polymeric matrices the so-called β-relaxation, related to rotation of polar group around C–C bonds, is typically observed in the nanosecond time scale.^58,59^ Moreover, the vibrational contribution, recently estimated for several matrices from *ab initio* vibrational intensities, accounts for approximately one unit of the dielectric constant and is definitely related to fast vibrational motions.^60^ Accordingly, we will separate the orientational reaction field due to polar solvation in matrices in two components: a dynamic component *F*_*or*_^dyn^ that, after photoexcitation, will readjust in response to the charge distribution in the dye in a time scale (say up to the first few nanoseconds) shorter than RISC and ISC processes; a second static component *F*_*or*_^st^ that will instead be considered frozen, at least in the time scale of interest. While showing different dynamics, and then affecting the time-resolved properties in different ways, at each instant of time the properties of the system are defined by the total reaction field *F*_*or*_ = *F*_*or*_^st^ + *F*_*or*_^dyn^.

**Figure 5:**
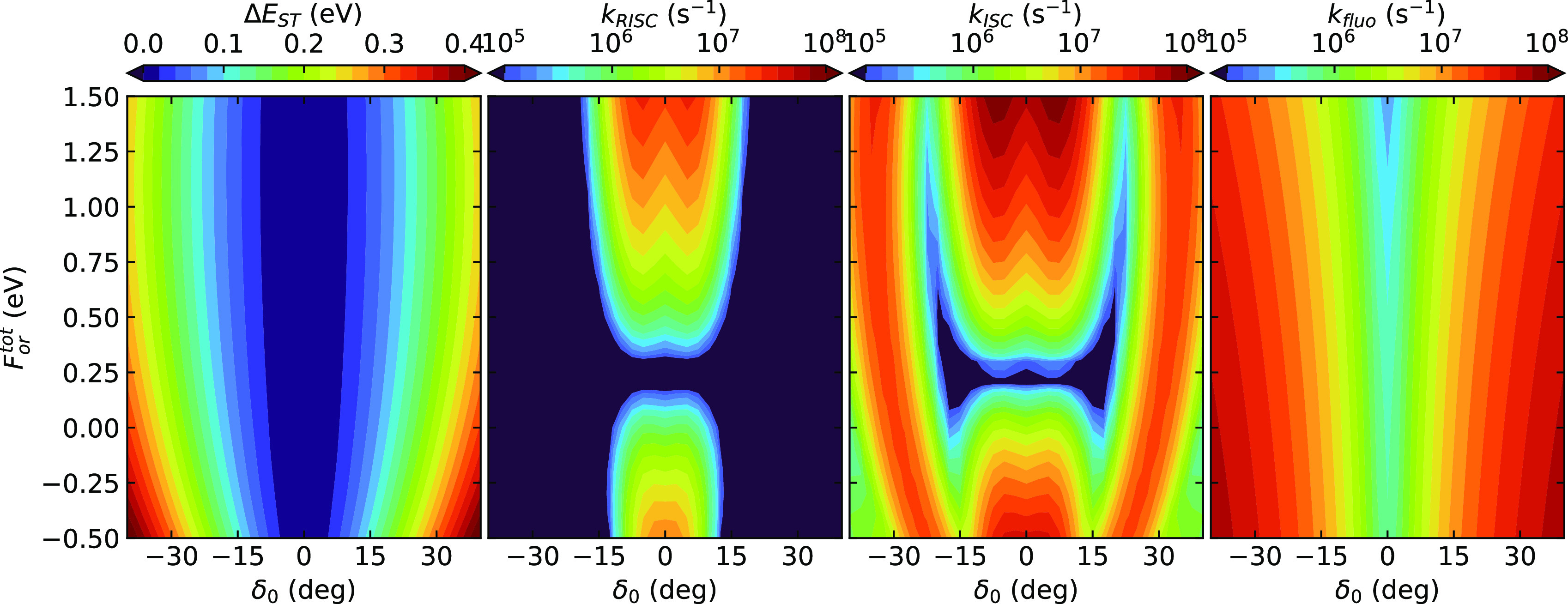
Color maps show as a function of *F*_*or*_ and δ_0_ the singlet triplet gap (leftmost panel) and in a logarithmic scale the calculated rates for ℏω_*c*_ = 4.0 × 10^–3^ eV.

*F*_*or*_- and δ_0_-dependent RISC and ISC rates are calculated following the same strategy discussed in the previous section, setting ϵ_*el*_ = 0.28 eV for all matrices. The radiative rate *k*_rad_ is finally calculated as a function of *F*_*or*_ as follows:^61^
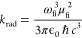
5where ω_*fi*_ and μ_*fi*_ are the transition frequency and dipole moment, respectively. The color maps in Figure 5 show the δ_0_ and *F*_*or*_ dependence of the singlet–triplet gap and of the rates of relevant processes. As expected, sizable RISC rates are only calculated in a narrow region of δ_0_ whose width varies with *F*_*or*_ but never extends beyond δ_0_ = ±20°. This region corresponds to the region where the fluorescence rate is minimal. The fluorescence rate shows a monotonic behavior vs *F*_*or*_, while both the RISC and ISC rates show a well-pronounced minimum at *F*_*or*_ ∼ 0.3 eV. The other rates entering the dynamical model in Figure 2 are set to constant values. Specifically, we set the non radiative rate as *k*_*nr*_ = 5 × 10^7^ s^–1^ and the phosphorescence rate as *k*_*ph*_ = 1 × 10^3^ s^–1^.

**Figure 6:**
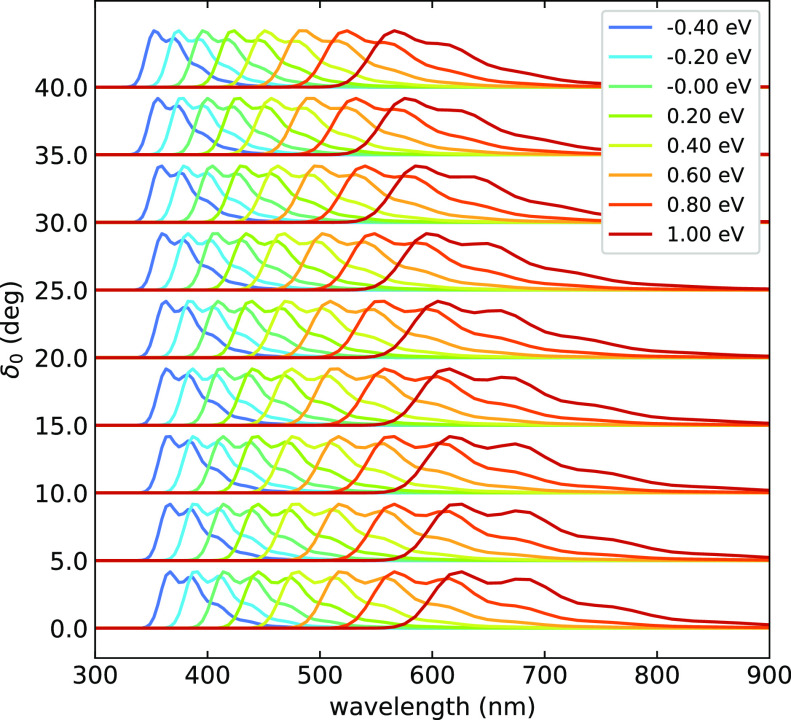
Normalized fluorescence spectra. In each row, referring to a different δ_0_ value, spectra calculated for different *F*_*or*_ are shown, color-coded as defined in the legend.

Figure 6 shows fluorescence spectra calculated for different *F*_*or*_ (according to the color code), each row corresponding to results obtained for different δ_0_. Normalized spectra are shown since *k*_rad_ in Figure 5 conveys information about the probability of the fluorescence process. The spectra markedly red-shift upon increasing *F*_*or*_, moving from ∼400 to ∼750 nm. This is of course due to the lowering of the energy of S_1_ state, an almost pure CT state, with the field. The concomitant widening of the band is simply related to the choice of showing the spectra against the wavelength; indeed, the band shape is marginally affected by *F*_*or*_ if the spectra are shown against energy (Figure S8). The dihedral angle has a smaller effect on the spectra than *F*_*or*_, but it largely affects the rates (cf. Figure 5).

We notice that neither the rates in Figure 5 nor the spectra in Figure 6 can be compared directly with experimental data. Indeed, either in solution or inside an organic matrix, conformational and dielectric disorder is present, so that to estimate rates and spectra one must average *F*_*or*_- and δ_0_-dependent results accounting for the relevant δ_0_ and *F*_*or*_ distributions. Specifically, to simulate the complex photophysics of DMAC-TRZ in matrices, we assume instantaneous excitation at *t* = 0 and calculate the time-resolved photophysics of the system as governed by the dynamical model in Figure 2. The calculation is tricky due to inhomogeneous broadening and even more due to the presence of a dynamic component of the reaction field.

**Figure 7:**
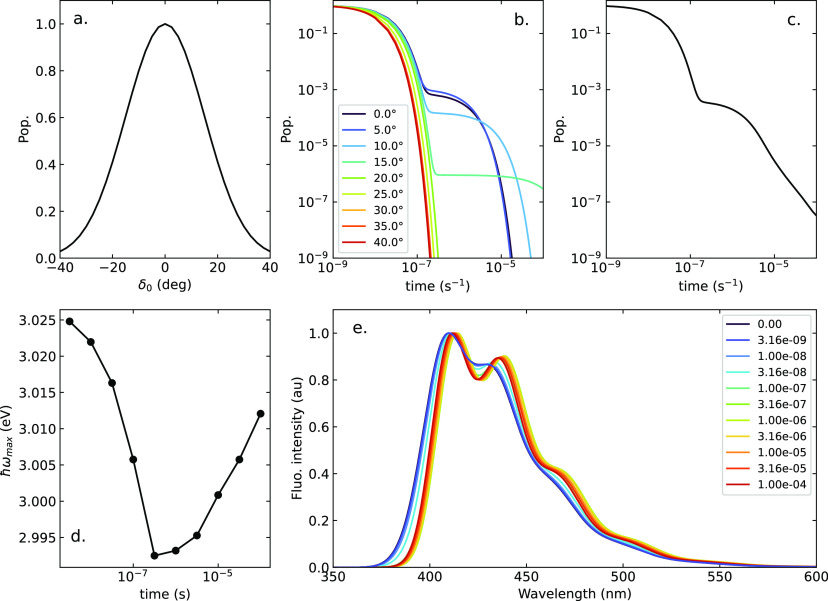
Photophysics of DMAC-TRZ in a hypothetical strictly nonpolar matrix: (a) the δ_0_ static Gaussian distribution, with standard deviation of σ = 15°; (b) the population of the singlet state (proportional to the fluorescence intensity) as a function of time, calculated for selected δ_0_ values; (c) the time evolution of the overall singlet state population; (d) the evolution with time of the frequency of the maximum of the fluorescence spectrum; and (e) time-resolved fluorescence spectra.

To start with, we consider a hypothetical, strictly nonpolar medium, where the orientational components of the reaction field vanish. Conformational disorder is then the only source of inhomogeneous broadening. The results in Figure 7 are obtained setting a Gaussian distribution of dihedral angles with a standard deviation σ = 15°. While arbitrary, this distribution compares favorably with the distribution of conformations obtained by a molecular dynamics simulations of DMAC-TRZ in an organic matrix.^57^ For comparison, Figure S10 shows results for a broader distribution.

Figure 7b shows the evolution of the singlet populations calculated for selected δ_0_ values. As expected, for large angles (δ_0_ > 15°) the singlet population decays with a single exponential, associated with prompt fluorescence. For smaller dihedral angles, the typical biexponential decay is observed, with delayed fluorescence showing up at long times. The largest RISC rates are seen at small angles; indeed, *k*_RISC_ values of similar magnitude are calculated for δ_0_ = 0 and 5°. The sizable *k*_RISC_ value calculated at δ_0_ = 0 (i.e., fully orthogonal D and A) strikingly contrasts with the widespread Marcus estimate of RISC rates. Indeed, the SOC matrix element connecting the singlet and triplet states vanishes at δ_0_ = 0°, that is, in the orthogonal conformation, so that the Marcus RISC rate should vanish there. However, the Marcus equation applies in the hypothesis that the SOC matrix element is independent of δ, while in DMAC-TRZ (and more generally in TADF dyes) it shows a large δ dependence. In these conditions, the non-Condon terms, neglected in the Marcus model, give a large contribution to RISC.^39^

Figure 7c shows the overall singlet population (proportional to the fluorescence intensity) calculated as a function of time, accounting for the initial Gaussian δ_0_ distribution shown in Figure 7a. Independent dynamics are calculated in each point on a grid in the δ_0_-distribution in Figure 7b and are then summed up accounting for the evolving singlet population in each point. The most striking result is the nonexponential decay at long times that is safely ascribed to the inhomogeneous broadening due to the static distribution of dihedral angle.

Figure 7e shows the corresponding time-dependent emission spectra. Here and in the following we show the *t* = 0 spectrum, that is, the spectrum calculated before any relaxation takes place. This spectrum is not experimentally accessible, but we show it as a reference to understand how large the effect of the relaxation is in the different environments. The spectra in Figure 7e show a resolved vibronic structure, as expected for an ideal nonpolar matrix. The spectra only marginal shift in frequency, in line with the minor effect of the dihedral angle on the position of fluorescence spectra, as best appreciated by the data in Figure 7d which shows how the maximum of the emission band evolves with time. Results for broader δ_0_ distributions are shown in Figure S10.

**Figure 8:**
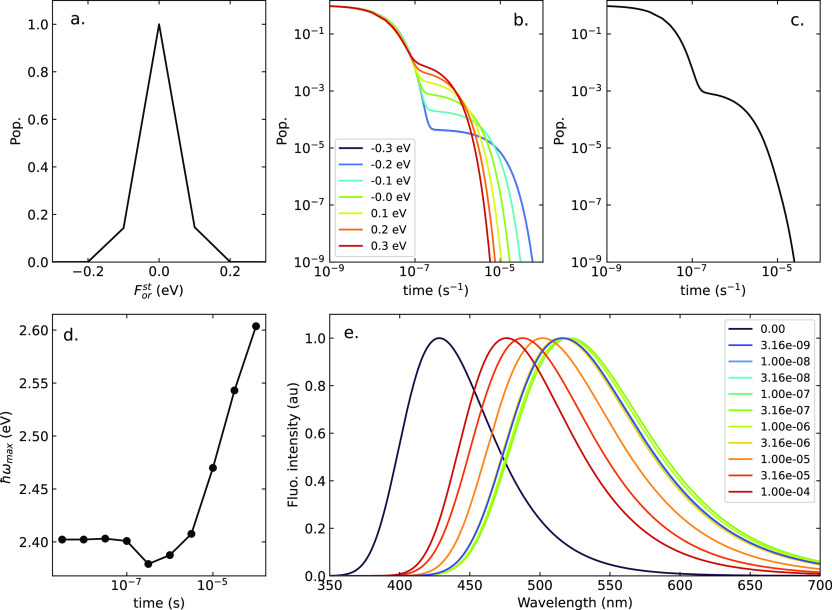
Photophysics of DMAC-TRZ in a hypothetical polar matrix with ϵ_*or*_^dyn^ = 0.25 eV and ϵ_*or*_^st^ = 0.05 eV and without conformational disorder δ_0_ = 0: (a) the static *F*_*or*_^st^ distribution; (b) the population of the singlet state as a function of time, calculated for selected *F*_*or*_^st^ values; (c) the time evolution of the overall singlet state population; (d) the evolution with time of the frequency of the maximum of the fluorescence spectrum; and (e) time-resolved fluorescence spectra.

Accounting for the dielectric disorder is trickier, as we have static and dynamic components. Figure 8 shows results for a hypothetical matrix with δ_0_ = 0 and no conformational disorder. As for the static dielectric contribution, we set a small value for the relevant relaxation energy, ϵ_*or*_^st^ = 0.05 eV: Figure 8a shows the corresponding *F*_*or*_^st^ distribution. RISC occurs from T_1_ and ISC from S_1_, but the *F*_*or*_ distributions relevant to the two states are very similar, so that for each *F*_*or*_^st^ we allow *F*_*or*_^dyn^ to relax to the equilibrium distribution for the triplet state and average the rates along this distribution. Then, for each *F*_*or*_^st^, we calculate the specific dynamics, as in Figure 8b (see the Supporting Information for further details). The first observation is that TADF becomes more efficient at large fields, even if the global effect of the orientational field is smaller than the effect of the angle. The resulting smaller inhomogeneous broadening reflects in a quasi-biexponential decay of the singlet-state population in Figure 8c. We may then conclude that the nonexponential tail seen at long times in the experimental emission intensity arises mainly from conformational disorder.

Because of dielectric disorder, time-resolved emission spectra in Figure 8e are broad and the vibronic structure is lost. The *t* = 0 spectrum, calculated before the relaxation of the dynamic component of the orientational field, has no experimental counterpart. The first experimentally accessible spectra, typically at few nanoseconds, are collected when the dynamical component of the dielectric field is partially or totally relaxed. Because of the lack of specific information about the time scale of the dielectric relaxation in matrices, the calculated spectra at *t* = 0 and *t* ∼ 3.16 × 10^–9^ s refer to a system where the dynamical component of the reaction field is unrelaxed and fully relaxed, respectively. The spectral shifts that follow are due to dielectric disorder. As shown in Figure 8d, minor shifts of the maximum of the emission band are observed during prompt fluorescence. A large red-shift is observed at the start of delayed fluorescence. The anomalous, very large blue-shift observed at long times is due to the dominance of states with a large negative electric field, showing a largely blue-shifted band.

We conclude that two sources of inhomogeneous broadening must be considered to understand the TADF photophysics in organic matrices: conformational disorder, which mainly governs the red-shift during prompt fluorescence and the appearance of a nonexponential decay tail, and static dielectric disorder, which mainly contributes to the spectral shifts during delayed fluorescence. The dynamic dielectric component instead mainly affects the position and shape of emission spectra.

#### Experimental Validation

**Figure 9:**
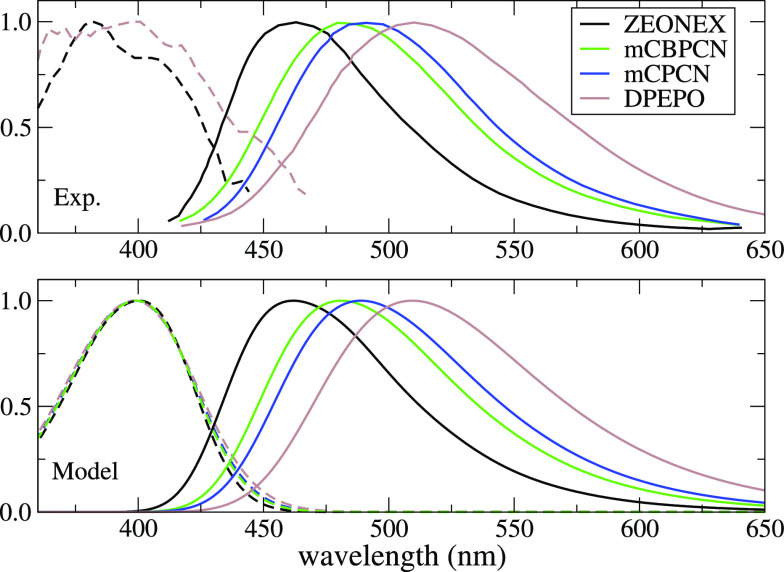
Top: experimental absorption (dashed lines) and emission spectra (continuous lines) of DMAC-TRZ in different matrices (from ref (34)). Bottom: theoretical absorption (dashed lines) and emission spectra (continuous lines) computed by using the solvent parameters as in the legend.

A detailed comparison with experiment requires specific estimates of matrix parameters. Because of the lack of dielectric relaxation data for relevant matrices, steady-state spectra of DMAC-TRZ give useful information. The top panel of Figure 9 shows steady-state absorption and emission spectra of DMAC-TRZ in different matrices (from ref (34)). Absorption spectra are marginally solvatochromic, as expected for a dye with a largely neutral ground state:^42,43,45^ because of the negligible dipole moment of the dye in the ground state, the reaction field distribution is centered around zero, irrespective of the solvent polarity. Instead, emission spectra progressively red-shift with increasing the matrix polarity from Zeonex to mCBPCN, mCPCN, and DPEPO. In DMAC-TRZ the delayed fluorescence represents a marginal fraction (<1%) of the emitted light, and steady-state spectra are dominated by prompt fluorescence (see Figure S6). The progressive red-shift of the emission band with increasing matrix polarity demonstrates that within the time window of prompt fluorescence the matrix readjusts at least partially in response to the variation of the dye polarity upon photoexcitation, lowering the excited state energy. Steady-state spectra in Figure 9 can then be fitted as in the bottom panel of Figure 9 to estimate ϵ_*or*_^dyn^ for each matrix, as shown in Table 3.

**Table 3:** Model Parameters for Organic Matrices Considered in This Work^a^

	ZEONEX	mCBPCN	mCPCN	DPEPO
ϵ_*el*_ (eV)	0.28	0.28	0.28	0.28
ϵ_*or*_^dyn^ (eV)	0.13	0.18	0.20	0.25
ϵ_*or*_^st^ (eV)	0.01	0.001	0.05	0.05
*k*_RISC_ (s^–1^)	9.4 × 10^4^	2.9 × 10^5^	4.4 × 10^5^	6.9 × 10^5^
*k*_RISC_^exp^ (s^–1^)	1.7 × 10^5^	9.3 × 10^5^	9.6 × 10^5^	1.1 × 10^6^

a Theoretical RISC rates from fitting of emission decays in Figures 10 and 11 following the procedure described in ref (62).

The parametrization of ϵ_*or*_^st^ is more delicate. The amount of static disorder depends on the matrix and its preparation (efficiency of packing, freedom to rearrange, amount of disorder, etc.) and on the specific emitter–matrix interaction (uniformity of the distribution of cavity shape and size).^10,63^ We therefore will introduce ϵ_*or*_^st^ as a free fitting parameter. In all spectra, we set the δ_0_ distribution as in Figure 7a. Results for a broader distribution are shown in the Supporting Information.

We start our discussion with results relevant to the Zeonex matrix in Figure 10. Zeonex is nonpolar, but as discussed above, a small dynamical contribution to ϵ_*or*_ must be introduced. We do not know the time scale of relevant dynamics, which may be comprised between a few picoseconds to a few nanoseconds, so we do not try to simulate the initial dynamics. The black curve in Figure 10, shown for reference, refers to the emission spectrum at time zero, that is, before any matrix relaxation. This spectrum is not experimentally accessible and only defines an upper limit for the early time emission spectrum. All other spectra are calculated by allowing the dynamical part of the dielectric field to rearrange to the relevant equilibrium distribution (see the Supporting Information for further details). Calculated spectra agree well with the experiment (cf. Figure S7),^34^ showing marginal frequency shifts in time, while the decay curve has the characteristic nonexponential tail.

**Figure 10:**
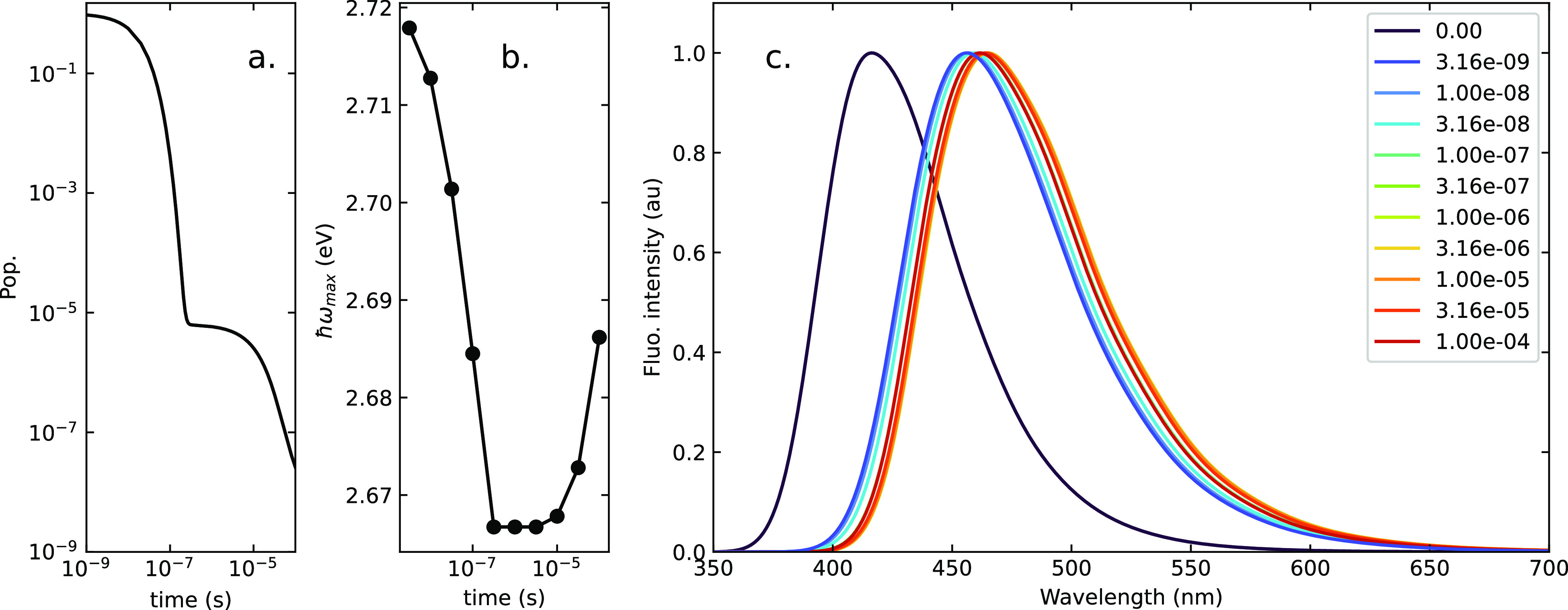
Simulation of the photophysics of DMAC-TRZ in the Zeonex matrix, assuming a Gaussian distribution for δ_0_ with σ = 15° (results for a wider distribution and for different ω_*c*_ are shown in Figures S15, S16, and S19): (a) time evolution of the singlet population, (b) time evolution of the maximum of the fluorescence spectra, and (c) time-resolved emission spectra. Time expressed in seconds.

**Figure 11:**
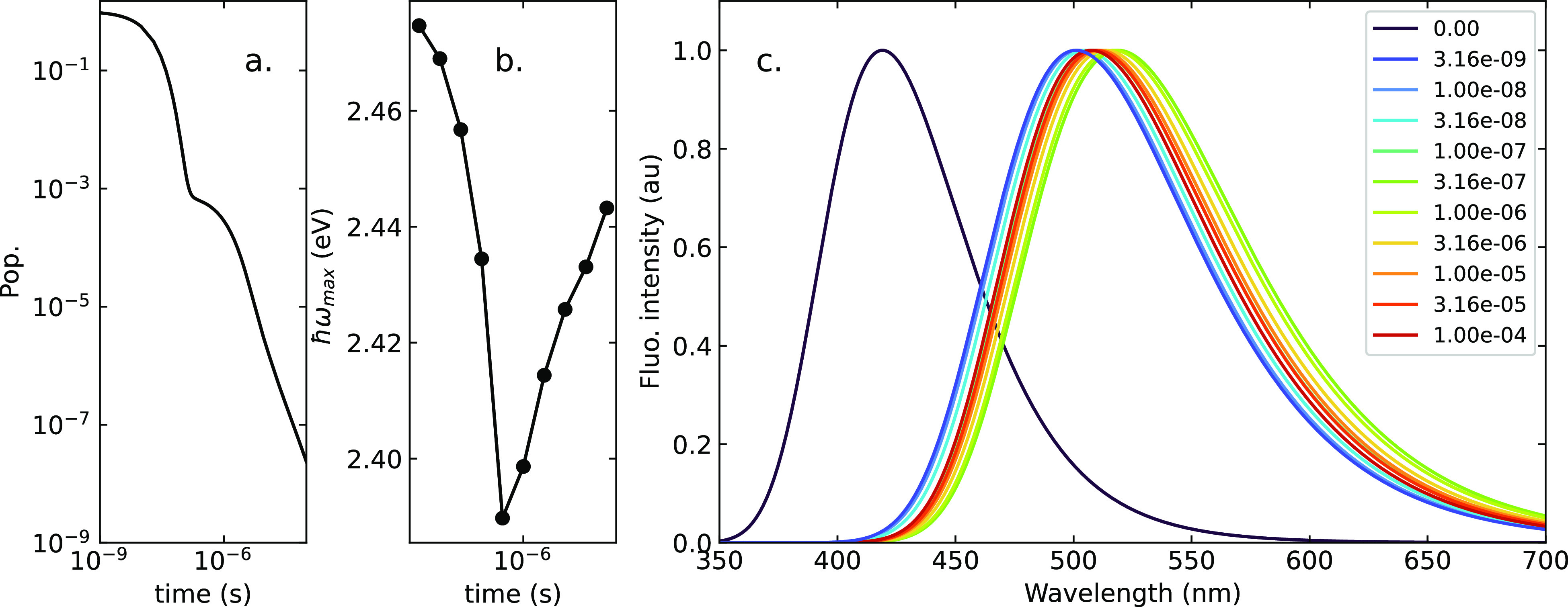
Simulation of the photophysics of DMAC-TRZ in the DPEPO matrix, assuming a Gaussian distribution for δ_0_ with σ = 15° (results for a wider distribution and for different ω_*c*_ are shown in Figures S17, S18, and S20): (a) time evolution of the singlet population, (b) time evolution of the maximum of the fluorescence spectra, and (c) time-resolved emission spectra. Time expressed in seconds.

Results for DPEPO matrix in Figure 11 also agree well with experiment (cf. Figure S7).^34^ The presence of a sizable static dielectric disorder is responsible for a sizable red-shift of the emission band, while the conformational disorder defines the nonexponential tail of the emission decay at long time.

The good agreement between calculated and experimental results collected at room temperature in nonpolar (Zeonex) and polar (DPEPO) matrices validates the model and specifically confirms the role of conformational and polar disorder in the definition of the intriguing spectral behavior of DMAC-TRZ in matrices. Similar results can be obtained in other matrices as well, but a detailed modeling of matrices requires a specific hypothesis on the distribution of the dihedral angle, which due to the lack of detailed information we maintain fixed in all calculations to a Gaussian centered at δ_0_ = 0 and σ = 15°. Similarly, we rely on an educated guess for the amount of static polar disorder. The amount of conformational and polar disorder actually depends not only on the specific matrix but also on the sample preparation, hindering a detailed modelization. Simulating temperature effects is also very delicate: polar and conformation disorder are affected by temperature, and the dielectric properties of the matrix itself are temperature-dependent.

## Conclusion

Focusing on DMAC-TRZ, a prototypical TADF dye, we proposed a comprehensive picture that rationalizes in a single unifying theoretical scheme the TADF photophysics of the dye dispersed in liquid solvents and in organic matrices of different polarity. RISC, ISC, and radiative rates are calculated in an original approach that fully accounts for the polarizability and polarity of the environment and for its rigidity. For the first time, we can accurately reproduce the highly nontrivial evolution of time-resolved emission spectra collected in diluted organic matrices in a wide time range after photoexcitation.

The molecular unit is described in terms of an essential state model, recently proposed, parametrized *ab initio* and validated against spectroscopic data in solution.^31^ The model crucially accounts for an effective molecular vibration, responsible for the vibronic structure, and for a conformational mode. The low-frequency conformational mode is responsible for thermal disorder and hence is a source of inhomogeneous broadening, with important effects of the intensity of absorption and emission spectra. The nonadiabatic treatment of vibrational and conformational modes is crucial to reliably calculate RISC and ISC rates and solves some of the critical issues that arise from the incorrect generalization of the Marcus model to systems where SOC shows a strong dependence on the conformational coordinate.^39^

The Onsager model, which offered the basis to address solvatochromism of polar dyes,^42,43,47^ is extended here to describe the interaction between the dye and its local dielectric environment. As recently discussed, polarizability and polarity effects are related to degrees of freedom with distinctively different time scales and must be treated accordingly.^48^ Polarizability, related to the fast electronic degrees of freedom of the medium, alters the molecular properties but is not a source of inhomogeneity. The environmental polarity, instead, associated with slow vibrational and conformational motions, is a powerful source of inhomogeneity. In liquid solvents, the dielectric relaxation is faster than prompt fluorescence and is easily addressed, with inhomogeneous broadening phenomena only showing up in the progressive broadening of spectral features with the solvent polarity.

In organic matrices the problem is much more complex. We discuss spectra collected in diluted samples, where we can neglect aggregation phenomena as well as self-absorption, also in view of the small extinction coefficient of DMAC-TRZ. Spectral diffusion due to energy transfer can also be excluded at these concentrations (estimated Förster rates are at least 2 orders of magnitude smaller than the singlet decay rate; see the Supporting Information). Optical spectra collected in these conditions then tell us most clearly that organic matrices do relax in the time scale of prompt fluorescence. Of course, this relaxation is only partial and is most probably slower than in liquid solvents, but the progressive red-shift of steady-state emission spectra with the matrix polarity (Figure 9) offers a clear indication in this direction.

More delicate are the two sources of static disorder, related to dielectric disorder in polar matrices and to conformational disorder. These two sources of disorder concur to define highly nontrivial inhomogeneous broadening phenomena in the TADF photophysics in organic matrices: the nonexponential tail of the emission decay at long delays is essentially due to conformational disorder. The complex temporal evolution of the spectra position and shape is due to the intertwined effect of static conformational and dielectric disorder.

Overall, the excellent agreement with experimental data suggests that the proposed model fully addresses the basic physics of TADF and can capture the subtle interplay between electronic, spin, and vibrational and conformational degrees of freedom of the molecule embedded in a polar and polarizable (partially) rigid matrix, as to explain subtle dynamical phenomena. This work sets a solid basis for a detailed modeling of TADF-OLED, offering reliable information about the variation of the RISC, ISC and fluorescence rates with the local environment and opening a new perspective about the need to account for static and dynamic conformational and dielectric disorder whose highly nontrivial effects must be properly addressed to govern the device behavior.

## Supporting Information Available

*Ab initio* parametrization of the molecular model, computational details, experimental steady-state and time-resolved spectra in solution, steady-state spectra in amorphous matrices, and additional results (PDF)
Video S1: simulation of time-resolved emission spectra of DMAC-TRZ in Zeonex related to Figure 10 (MPG)
Video S2: simulation of time-resolved emission spectra of DMAC-TRZ in DPEPO related to Figure 11 (MPG)

## Supplementary Material

ja2c05537_si_001.pdf

ja2c05537_si_002.mpg

ja2c05537_si_003.mpg
