# Supplementary material for: Thermally Activated Delayed Fluorescence: Polarity, Rigidity, and Disorder in Condensed Phases
Source: J Am Chem Soc. 2022 Aug 9;144(33):15211–22. doi: 10.1021/jacs.2c05537 (PMC9413221; doi:10.1021/jacs.2c05537)
Supplement: Supplementary file 1 — ja2c05537_si_001.pdf [file ja2c05537_si_001.pdf]

Supporting Information: Thermally activated delayed fluorescence: polarity, rigidity and disorder in condensed phases

D. K. Andrea Phan Huu,† Sangeeth Saseendran,† Rama Dhali,† Larissa Gomes Franca,‡ Kleitos Stavrou,‡ Andrew Monkman,*,‡ and Anna Painelli*,†

†*Department of Chemistry, Life Sciences and Environmental Sustainability, University of Parma, 43124 Parma, Italy.*

‡*Department of Physics, Durham University, South Road, DH1 3LE, UK*

E-mail: a.p.monkman@durham.ac.uk; anna.painelli@unipr.it

The ab-initio parametrization of the molecular model

The parametrization of the molecular model in Eq. 1 is performed in Ref. S1 against results of TD-DFT calculations (M06-2X functional, 6-31G(d) basis set, Tamm-Dancoff approximation). Starting from the ground state equilibrium geometry we perform a rigid scan analysis varying the dihedral angle δ and calculate the energy of the ground state, of the lowest singlet (S_1) and of the lowest triplet state (T_1). A best fit of the potential energy curves allows to extract the electronic parameters and their δ-dependence. Specifically we estimate z, τ_0, k, β_0, ω_c and a. The coupling to the high frequency molecular vibration ϵ_v is obtained summing up the vibrational relaxation energies calculated upon ionization of the DMAC and TRZ fragments. The vibrational energy is extracted to best fit optical spectra, and marginally affects the results. The spin-orbit coupling matrix elements V_{SOC} and W_{SOC} are

obtained from the fit of the δ-dependent spin orbit coupling between S_1 and T_1 and between the ground state and T_1 (SOC coupling are obtained in Orca 4.1 package. Finally, the dipole moment associated to the zwitterionic state μ_0 is obtained from the slope of the transition energy from the ground to S_1 state.

The solvation model

The fast component of the reaction field is dealt with in the antiadiabatic approximation leading to a renormalization of the model Hamiltonian with the reduction of the gap between the zwitterionic and the neutral states, $2z \to 2z - \epsilon_{el}$. A closed expression for ϵ_{el} can be obtained assuming that the dye occupies inside the medium a spherical cavity of radius r_0:[S2,S3]

$$\epsilon_{el} = \frac{\mu_0^2}{4\pi\varepsilon_0 r_0^3} \frac{\eta^2 - 1}{2\eta^2 + 1} \tag{1}$$

In organic media the refractive index spans a narrow range (1.4-1.5 in organic solvents, 1.5-1.7 in organic matrices), leading to a narrow variability for $\epsilon_{el} \sim 0.24\,\text{eV}$ to $0.30\,\text{eV}$ (estimated as the Onsager radius (increased by 0.5 Å) $r_0 \sim 6.44\,\text{Å}$.[S4]

The second component of the reaction field is associated with the vibrational and orientational motions of polar molecules in the medium. Relevant degrees of freedom are treated in the adiabatic approximation (see main text). Polar solvation is described by the solvent relaxation energy, ϵ_{or}: S5

$$\epsilon_{or} = \frac{\mu_0^2}{4\pi\varepsilon_0 r_0^3} \left(\frac{\epsilon_{st} - 1}{2\epsilon_{st} + 1} - \frac{\eta^2 - 1}{2\eta^2 + 1} \right) \tag{2}$$

where ϵ_{st} is the static dielectric constant of the medium.

Ground and excited state F_{or} distribution

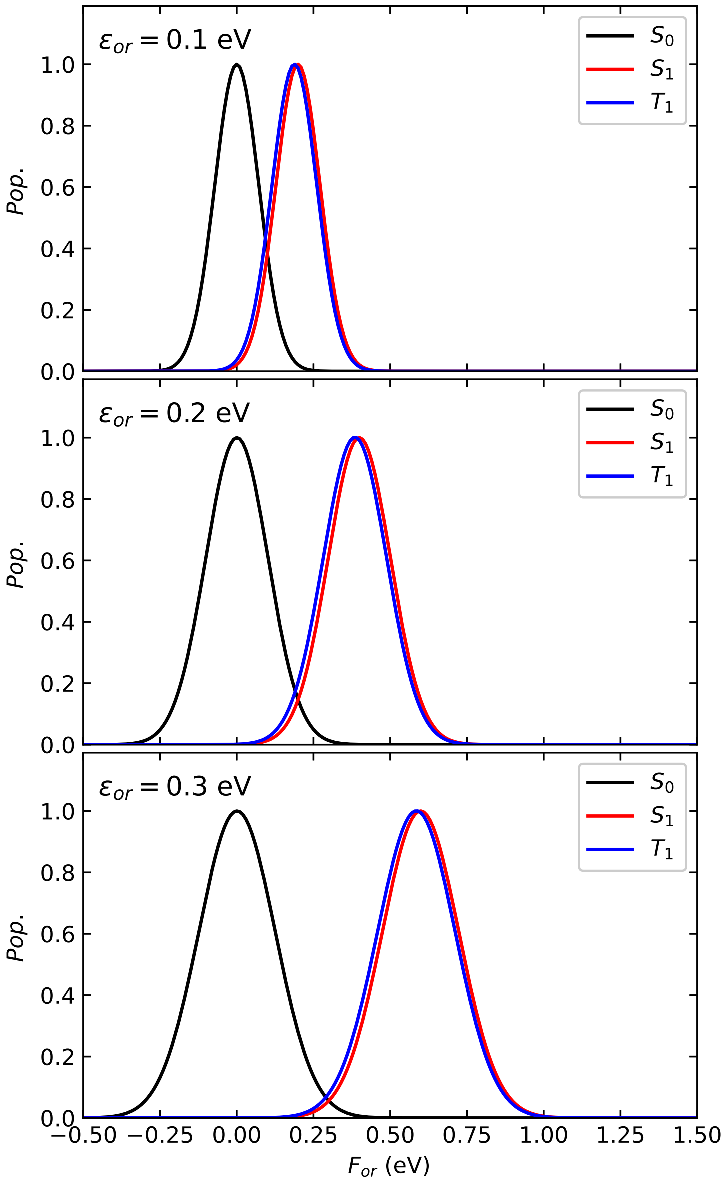

Figure S1: F_{or}-dependent distributions of S_0, S_1 and T_1 states of DMAC-TRZ (parameters from Table 1) in hypotetical solvents with $\epsilon_{el} = 0$ and ϵ_{or} as specified in each panel.

The adiabatic calculation of optical spectra

Steady state absorption and fluorescence spectra in Fig. 3 are obtained via a fully non-adiabatic calculations, i.e. treating both the molecular vibration and the conformational modes non-adibatically. Exactly the same results can be obtained treating the conformational mode adiabatically, as in Ref. S1. In short, we neglect the kinetic energy associated with the conformational mode and solve the molecular Hamiltonian on a grid of F_{or} and δ values. The molecular Hamiltonian accounts for the electronic and vibrational degrees of freedom, so that the relevant basis is the direct product of the electronic states times the first M states of the harmonic oscillator associated to the vibrational coordinate. M is large enough (typically in this work M = 10) to ensure convergence. The Hamiltonian matrix is then diagonalized numerically to get vibronic eigenstates. Absorption and fluorescence spectra are computed assigning to each transition a Gaussian bandshape with a half-width at half-maximum of G = 0.08 eV. Spectra calculated for different For and d values are then summed over accounting for their Boltzmann weight with reference to the energy of the ground state for absorption spectra, and of the lowest singlet state for fluorescence spectra, respectively.

Liquid solvents: calculation of the δ-distributions

the Fig. S2 displays the area normalized population of the S_0, S_1 and T_1 states at 300 K as a function of δ for different values of the external field, calculated in liquid solvents. Distributions can be computed in two ways, accounting for δ either as a adiabatic or non-adiabatic coordinate. The distributions computed accounting for δ in the adiabatic approximation is obtained as:

$$P_i(\delta) = \frac{\exp\left(-\frac{E_i(\delta)}{k_b T}\right)}{\int_{-\infty}^{+\infty} d\delta \exp\left(-\frac{E_i(\delta)}{k_b T}\right)} \tag{3}$$

where $E_i(\delta)$ is the potential energy surface, with i running on S_0, S_1 and T_1, the lowest vibronic eigenstates of each respective manifold.

When δ is accounted for as a non-adiabatic mode, a generic eigenfunction k of eq. 2 is:

$$\Psi_k(r, Q, \delta) = \sum_{i,v,p} c_{kivp}\phi_i(r)\chi_v(Q)f_p(\delta) \tag{4}$$

where c_ivp is the coefficient. $\phi_i(r)$ is an electronic basis function and $\chi_v(Q)$ ($f_p(\delta)$) is the vibrational wave function associated to the Q (δ) mode.

The non-adiabatic distributions (dotted lines) are computed as:

$$P(\delta) = \sum_k p_k \sum_{ivpp'} c^*_{kivp}c_{kivp'}f^*_{ivp}(\delta)f_{ivp'}(\delta) \tag{5}$$

where $p_k = \exp\left(\frac{E_k}{k_bT}\right) / \sum_k \exp\left(\frac{E_k}{k_bT}\right)$ is the population of the k-th non-adiabatic eigenstate in the manifold of interest.

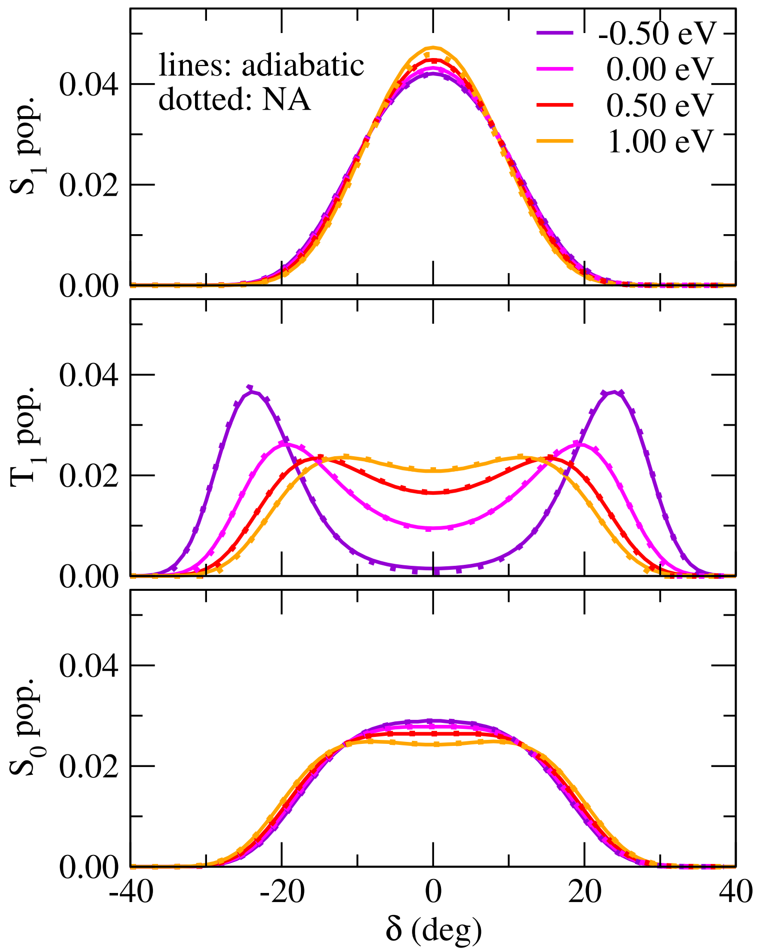

Figure S2: Distributions as a function of δ of the S_1, T_1 and S_0 states, accounting for δ as a adiabatic mode (continuous lines) and a non-adiabatic mode (dotted lines), for different values of an external field.

Experimental data

DMAC-TRZ in solution

Degassed solutions of DMAC-TRZ for photophysical characterisation were prepared at 0.8 mM concentration in methylcyclohexane (MCH), toluene (PhMe), and chloroform (CHCl3). Degassed solutions were obtained by 5 freeze-pump-thaw cycles to remove all dissolved oxygen. Steady state emission spectra in Fig. S3 were acquired using a Horiba Jobin Yvon Fluorolog-3 spectrofluorometer. Time-resolved photoluminescence spectra in Fig. S4 and the luminescence decay profiles in Fig. S5 were recorded using an ultra-fast 4 PICOS iCCD camera (Stanford Computer Optics) with a pulsed (10 Hz) Nd:YAG laser (EKSPLA-SL312) excitation source at 355 nm.

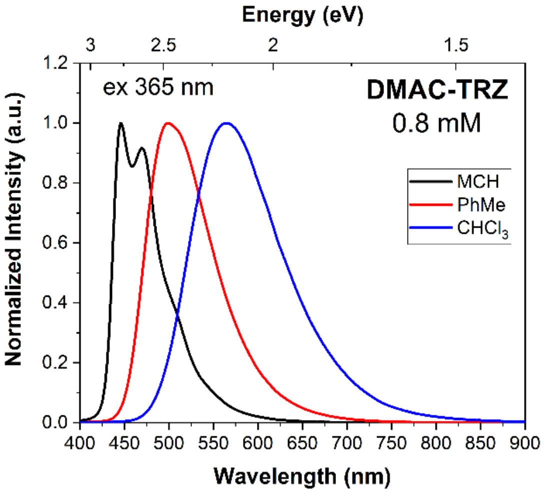

Figure S3: Photoluminescence (PL) spectra of DMAC-TRZ in different solvents at 0.8 mM concentration. All measurements were performed at room temperature, using a 365 nm excitation source.

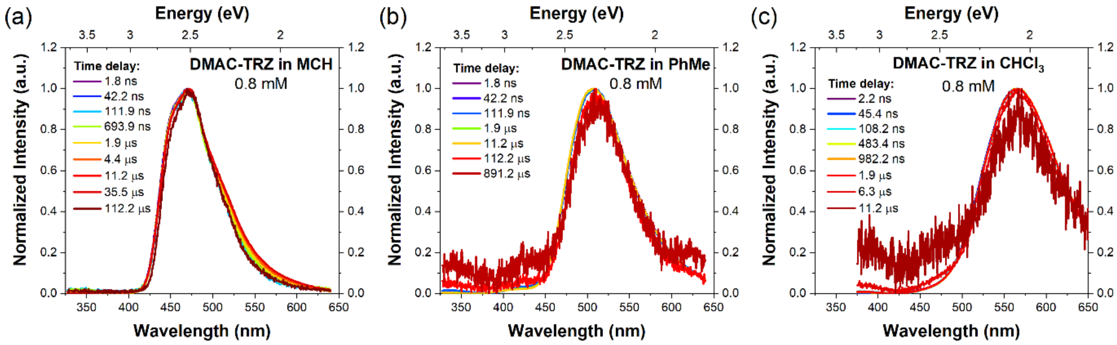

Figure S4: Time resolved emission spectra of DMAC-TRZ in (a) MCH, (b) PhMe and (c) CHCl3 solutions. All measurements were performed in degassed solutions at room temperature, using a 355 nm excitation source.

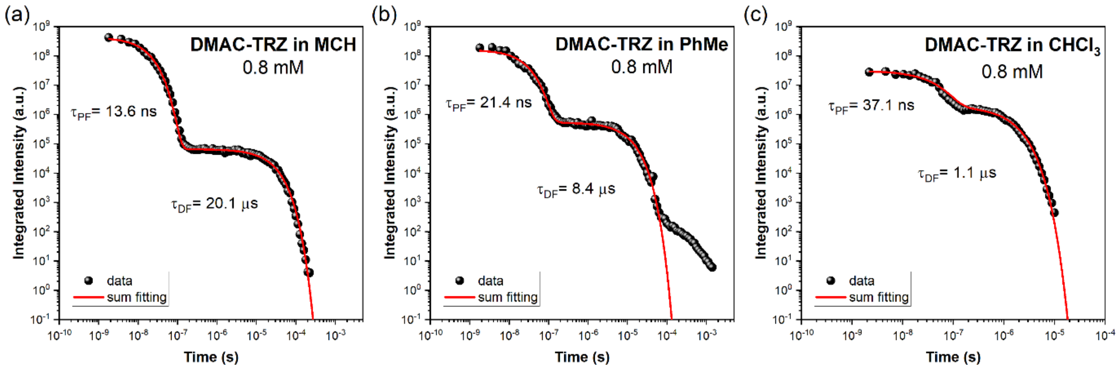

Figure S5: Kinetic decays of DMAC-TRZ in (a) MCH, (b) PhMe and (c) CHCl3 solutions, at 0.8 mM concentration. All measurements were performed in degassed solutions at room temperature, using a 355 nm excitation source. The data are fitted using a biexponential function.

DMAC-TRZ in organic matrices

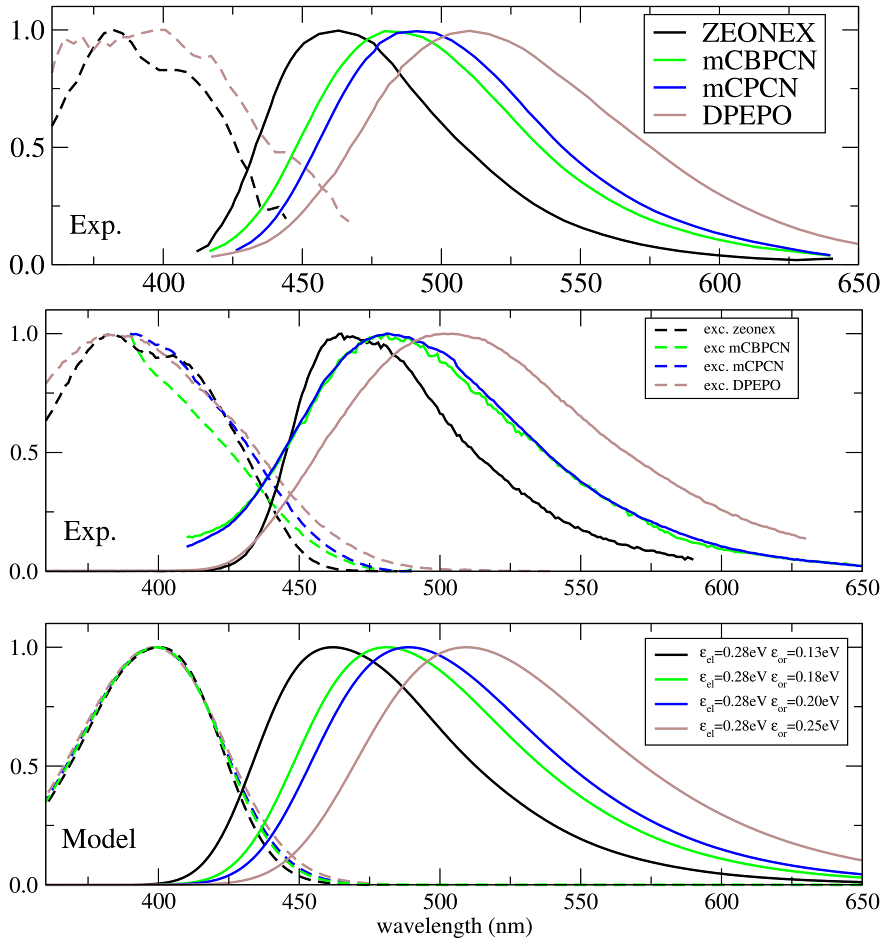

Figure S6: Top panel: experimental absorption (dashed lines) and emission spectra (continuous lines) of DMAC-TRZ in different matrices from Ref. S6. Middle panel: experimental excitation (dashed lines) and emission spectra (continuous lines) measured in ambient conditions. Bottom panel: theoretical absorption (dashed) and emission (continuous) spectra computed using the solvent parameters as in legend. The marginal differences between experimental emission spectra collected in degassed (top) and ambient conditions (middle) can be ascribed to a contribution from the host material.

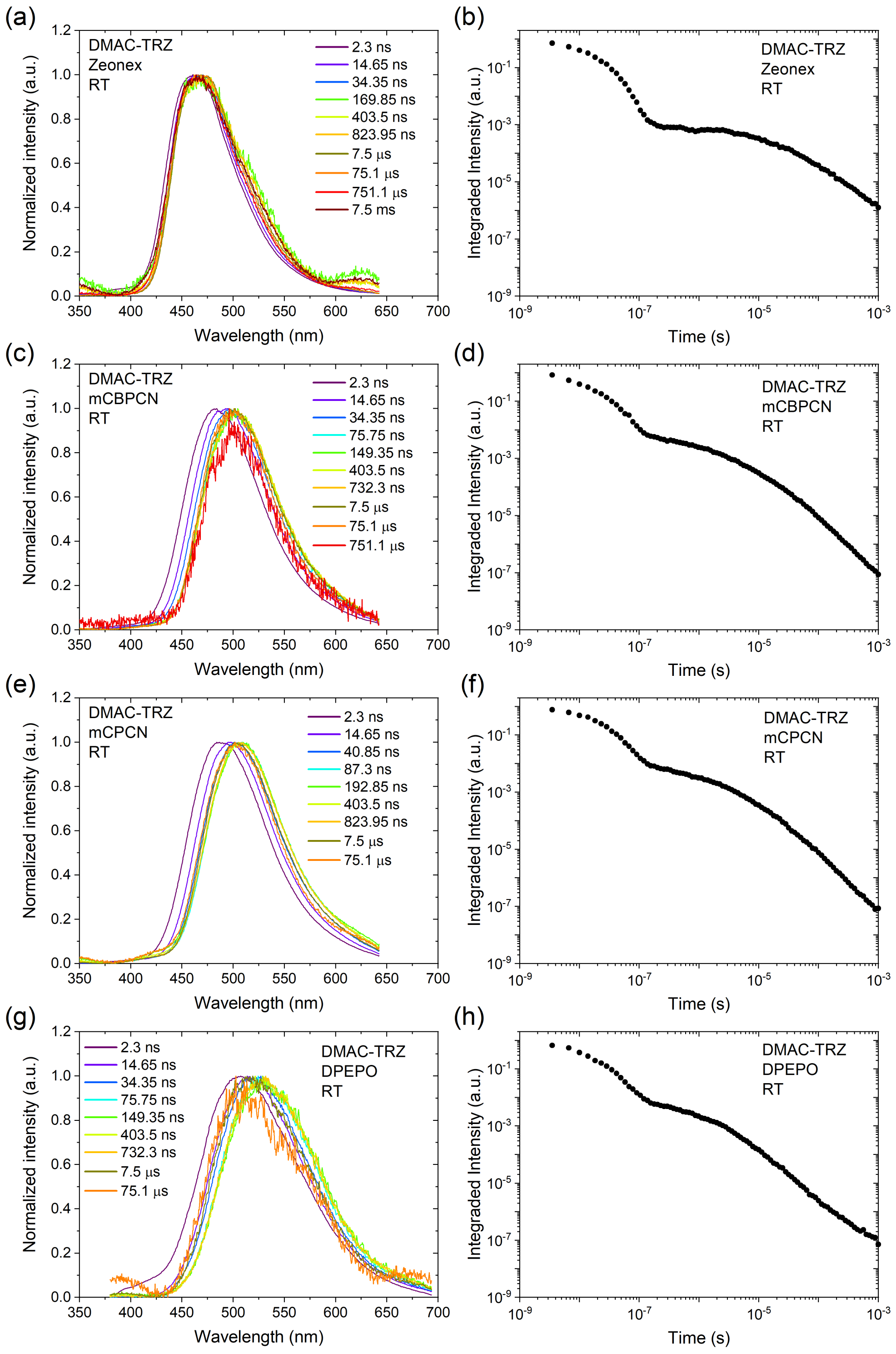

Figure S7: Room temperature experimental time-resolved emission (left column) and emission decay (right column) of DMAC-TRZ in different matrices at 1% w/w concentration from Ref. S6.

Estimate of the resonant energy homotransfer rate in matrices with dye loading 1%

Derivation of k_{RET}

The rate of energy transfer from the energy donor, $\mathcal{D}$, to the energy acceptor, $\mathcal{A}$, in the weak coupling regime is given by Fermi's golden rule:

$$k_{RET} = \frac{1}{\hbar^2} V^2 \delta(\nu_{\mathcal{D}} - \nu_{\mathcal{A}}) \tag{6}$$

where the coupling term V can be expressed as:

$$V = \frac{e^2}{4\pi\epsilon_0\eta^2} \left\langle \phi_{\mathcal{D}^*}(1)\phi_{\mathcal{A}}(2) \right| \frac{1}{r_{\mathcal{D}\mathcal{A}}} \left| \phi_{\mathcal{D}}(1)\phi_{\mathcal{A}^*}(2) \right\rangle \tag{7}$$

$$V = \frac{1}{4\pi\epsilon_0\eta^2} \frac{|\vec{\mu}_{\mathcal{D}}| \cdot |\vec{\mu}_{\mathcal{A}}|}{R^3_{\mathcal{D}\mathcal{A}}} \kappa \tag{8}$$

In the first line $\phi_{\mathcal{D}}$ ($\phi_{\mathcal{A}}$) is the unperturbed wavefunction of $\mathcal{D}$ ($\mathcal{A}$), the asterisk marking the excited species, and $r_{\mathcal{D}\mathcal{A}}$ is the distance between $\mathcal{D}$ and $\mathcal{A}$. The second line is obtained adopting the dipole approximation. $\mu_{\mathcal{D}}$ ($\mu_{\mathcal{A}}$) is the transition dipole moment of $\mathcal{D}$ ($\mathcal{A}$), $R_{\mathcal{D}\mathcal{A}}$ is the distance between the dipoles and κ is the orientational factor.

The energy conservation term is rewritten as:

$$\delta(\nu_{\mathcal{D}} - \nu_{\mathcal{A}}) = \int d\nu \delta\left(\nu_{\mathcal{D}} - \nu\right) \delta\left(\nu - \nu_{\mathcal{A}}\right) \tag{9}$$

$$\delta(\nu_{\mathcal{D}} - \nu_{\mathcal{A}}) = \int d\nu F(\nu) A(\nu) \tag{10}$$

where in the second line the delta functions are substituted with the area-normalized absorption of the acceptor ($A(\nu)$) and fluorescence of the donor ($F(\nu)$).

In homotransfer, $\mu_t = \mu_{\mathcal{D}} = \mu_{\mathcal{A}}$, so that the resonance energy transfer rate is given by:

$$k_{RET} = \frac{\kappa^2}{1600c\pi^2\varepsilon_0^2\hbar^2\eta^4}\frac{\mu_t^4}{R^6}\int d\tilde{\nu}F(\tilde{\nu})A(\tilde{\nu}) \quad (11)$$

where $\kappa^2 = 2/3$ is the squared orientational factor for random orientation of molecules, η is the refractive index, μ_t is the transition dipole moment, R is the intermolecular distance and the overlap integral is computed on area-normalized absorption and emission spectra (integration over the wavenumber, $\tilde{\nu}$).

We estimate $R \sim 4 \times 10^{-9}$ m for DMAC-TRZ at 1% w/w concentration in DPEPO, the value for other matrices (same concentration) is similar. The transition dipole moment is estimated from the radiative lifetime τ_R in nanoseconds as follows:[S7]

$$\frac{1}{\tau_R} = \frac{8\pi\tilde{\nu}^3\mu_t^2}{3000\hbar\epsilon_0} \quad (12)$$

where $\tilde{\nu}$ is the wavenumber of the emission (cm^{-1}). All other values enter in SI units.

The radiative lifetime is obtained as the ratio of the experimental lifetime τ and the fluorescence quantum yield, Φ. Data relevant to cyclohexane solution in Ref. S1 have $\Phi = 0.22$, $\tau = 45$ ns, leading to $\mu_t = 2.5$ D. Results have minor dependence on solvent: ~1.8 D in chloroform and ~ 2.4 D in toluene. The overlap O is computed from absorption and emission spectra of DMAC-TRZ in cyclohexane in order to account for the maximum overlap.

With these data, the estimated upper limit for RET rate, $k_{ret} \sim 4.6 \times 10^5\,s^{-1}$, is several orders of magnitude smaller than the fluorescence rate, so that the homo-transfer may be neglected in diluted samples.

Simulation of time resolved emission spectra

To compute time-resolved emission spectra in organic matrices a grid is defined on the $\{F_{or}^{st}, \delta_0\}$ plane (with uniform spacing $\Delta F_{or}^{st} = 0.10\,\mathrm{eV}$ and $\Delta\delta_0 = 2.5°$). In each point of the grid, the time-dependent populations of S_0, S_1 and T_1 states are calculated integrating the dynamical equations (fourth order Runge-Kutta method):

$$\begin{cases} [\dot{S_1}] = -\left(k_{nr} + k_{fluo}\right)[S_1] + k_{RISC}\,[T_1] \\ [\dot{T_1}] = k_{ISC}\,[S_1] - \left(k_{RISC} + k_{phospho}\right)[T_1] \\ [\dot{S_0}] = \left(k_{fluo} + k_{nr}\right)[S_1] + k_{phospho}\,[T_1] \end{cases} \tag{13}$$

where in each point of the grid the rates are obtained as thermal averages over the F_{or}^{dyn} distribution.

Time-resolved emission spectra are then obtained summing relevant (F_{or} and δ_0 dependent) spectra weighted for the instantaneous population of the S_1 state.

Miscellaneous results

Table S1: Computed k_{RISC} values (s^{-1}) of DMAC-TRZ in amorphous matrices for different values of $\hbar\omega_c$

$\hbar\omega_c$ (eV)	Zeonex	mCBPCN	mCPCN	DPEPO
2×10^{-3}	7.9×10^4	2.4×10^5	3.8×10^5	5.9×10^5
4×10^{-3}	9.4×10^4	2.9×10^5	4.4×10^5	6.9×10^5
8×10^{-3}	1.2×10^5	3.4×10^5	4.9×10^5	7.4×10^5

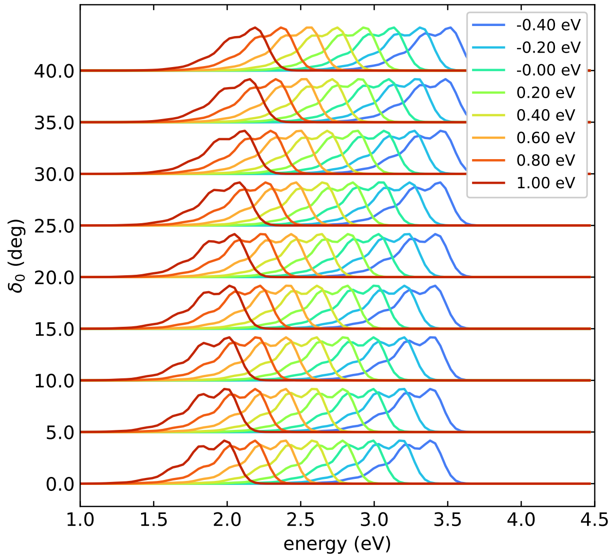

Figure S8: Normalized fluorescence spectra. In each row, referring to a different δ_0 value, spectra calculated for different F_{or} are shown, colour-coded as defined in the legend.

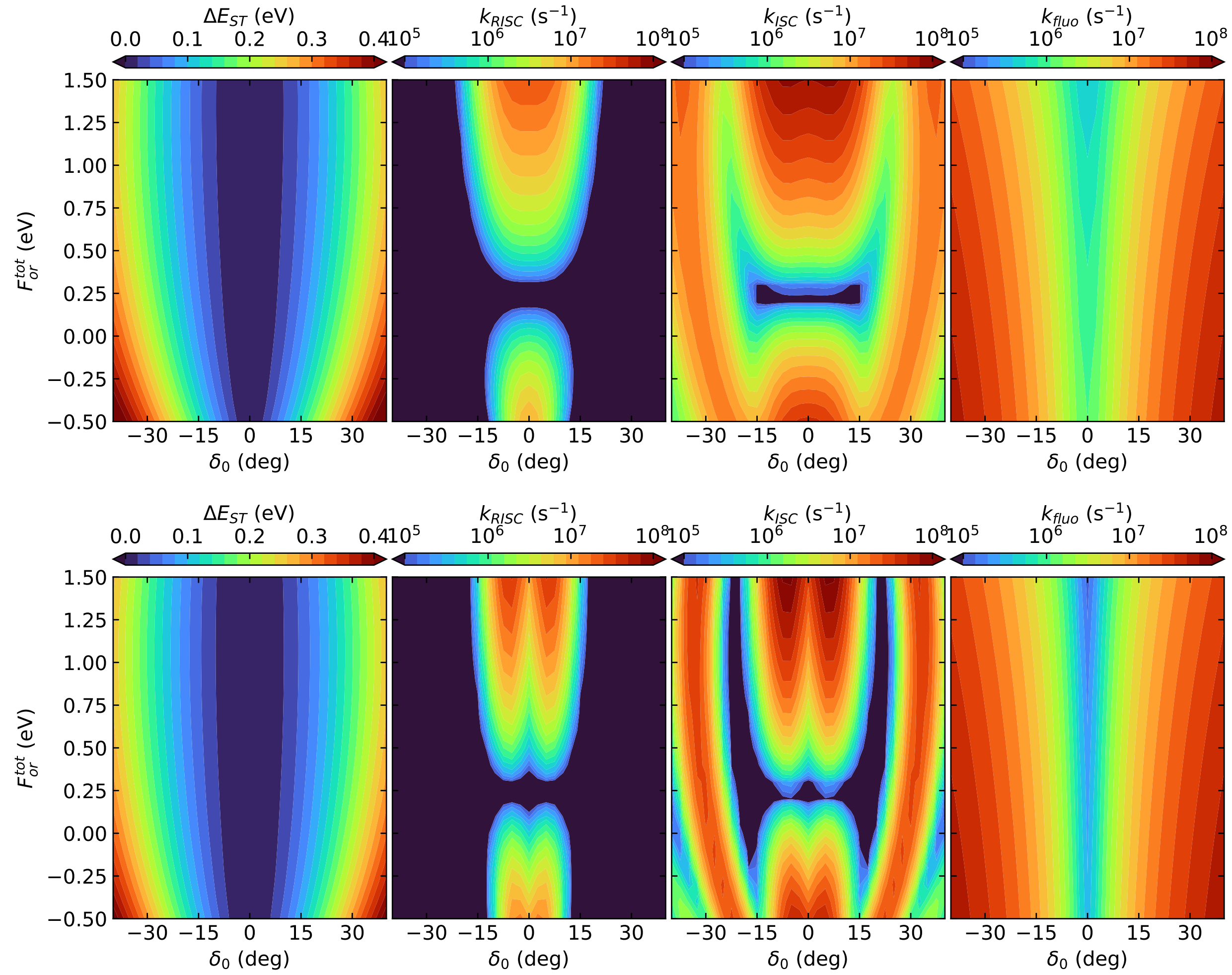

Figure S9: The color maps show as a function of F_{or} and δ_0 the singlet triplet gap (leftmost panel) and in a logarithmic scale the the calculated rates for $\hbar\omega_c = 2.0 \times 10^{-3}$ eV (top) and $\hbar\omega_c = 8.0 \times 10^{-3}$ eV (bottom). To be compared with results in Fig. 5 for $\hbar\omega_c = 4.0 \times 10^{-3}$ eV.

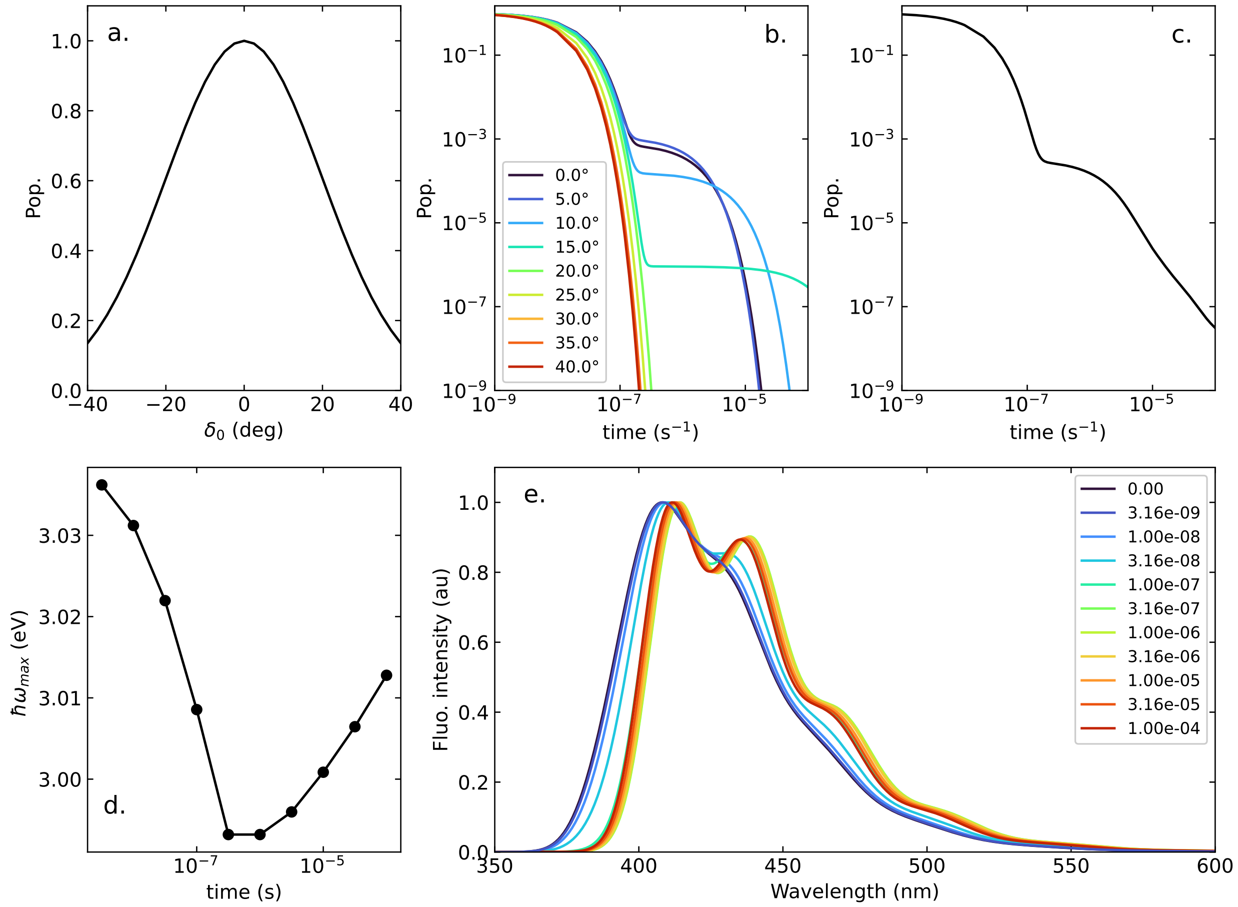

Figure S10: Same as Fig. 7, but using a broader distribution ($\sigma = 20°$) to simulate a higher degree of conformational disorder.

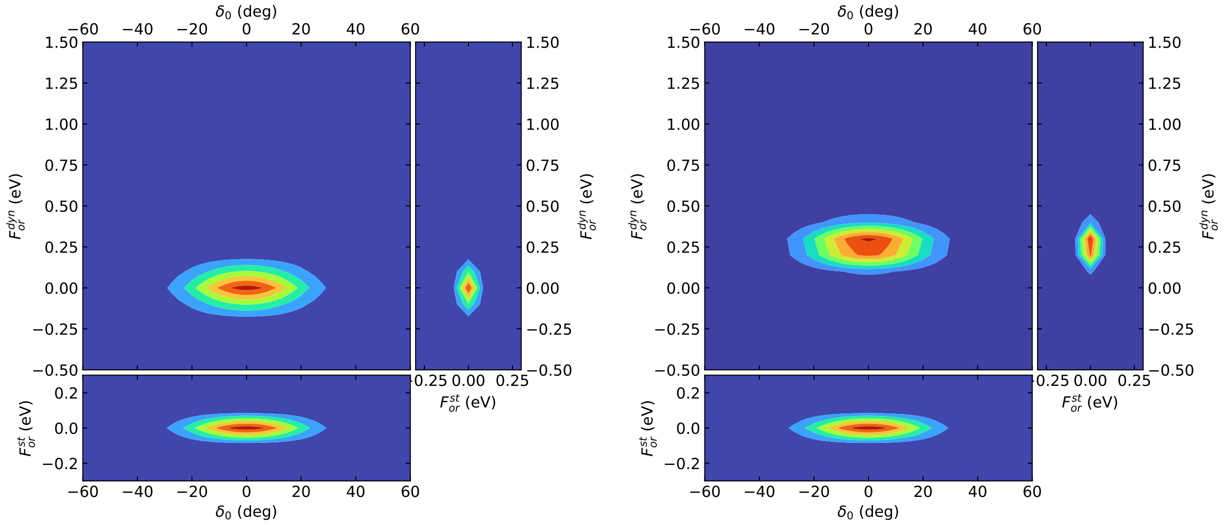

Figure S11: Projection of the S_1 population of DMAC-TRZ in Zeonex on the planes spanned by the δ_0, F_{or}^{st} and F_{or}^{dyn} coordinates. Left: immediately after excitation. Right: after the dielectric relaxation along F_{or}^{dyn}.

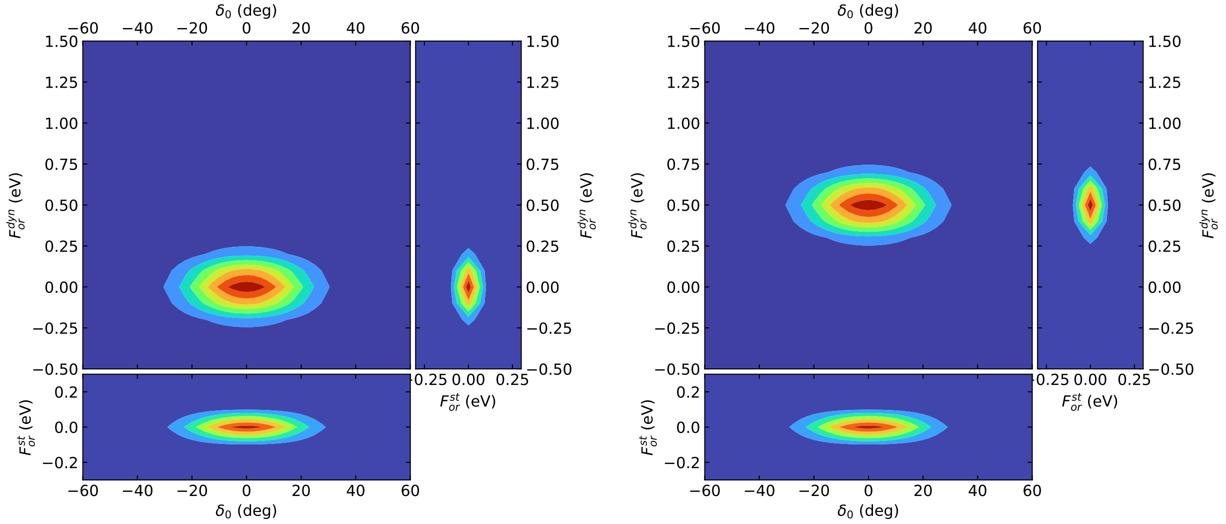

Figure S12: Projection of the S_1 population of DMAC-TRZ in DPEPO on the planes spanned by the δ_0, F_{or}^{st} and F_{or}^{dyn} coordinates. Left: immediately after excitation. Right: after the dielectric relaxation along F_{or}^{dyn}.

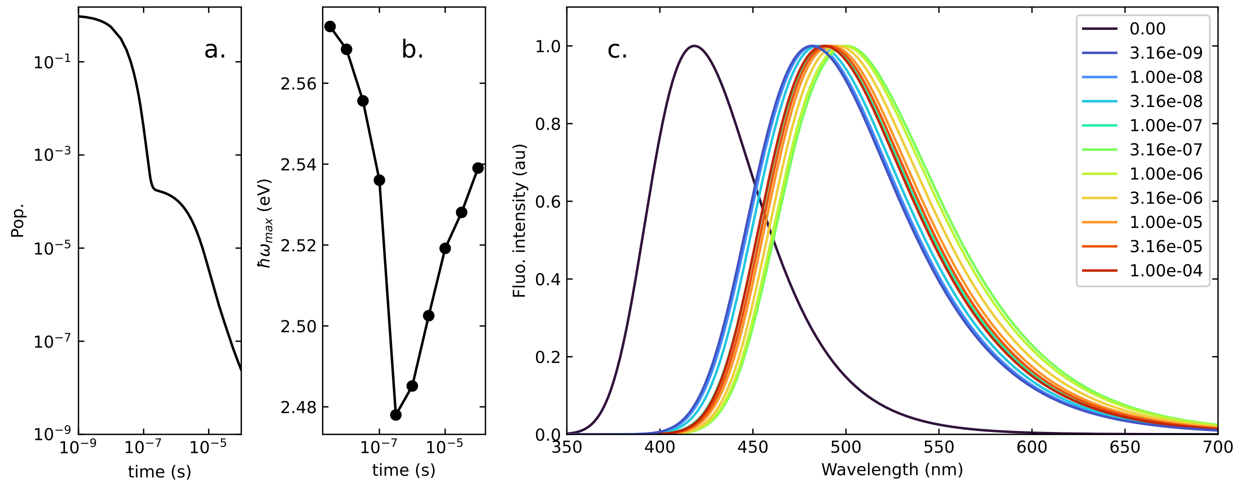

Figure S13: Simulation of the photophysics of DMAC-TRZ in mCPCN matrix. (a) time-evolution of the singlet population (b) time-evolution of the maximum of the fluorescence spectra (c) time-resolved emission spectra. Time expressed in seconds.

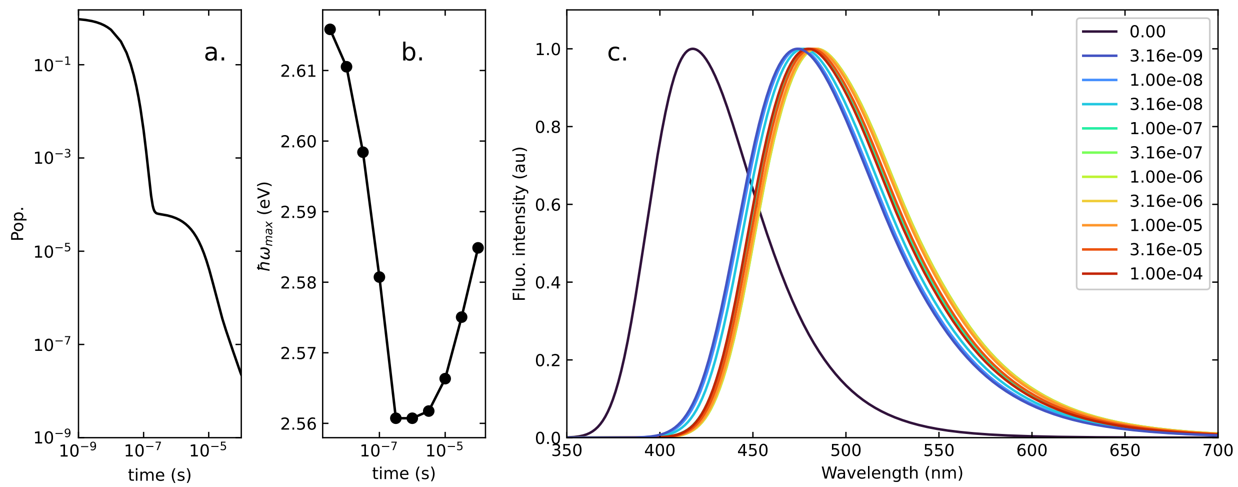

Figure S14: Simulation of the photophysics of DMAC-TRZ in mCBPCN matrix. (a) time-evolution of the singlet population (b) time-evolution of the maximum of the fluorescence spectra (c) time-resolved emission spectra. Time expressed in seconds.

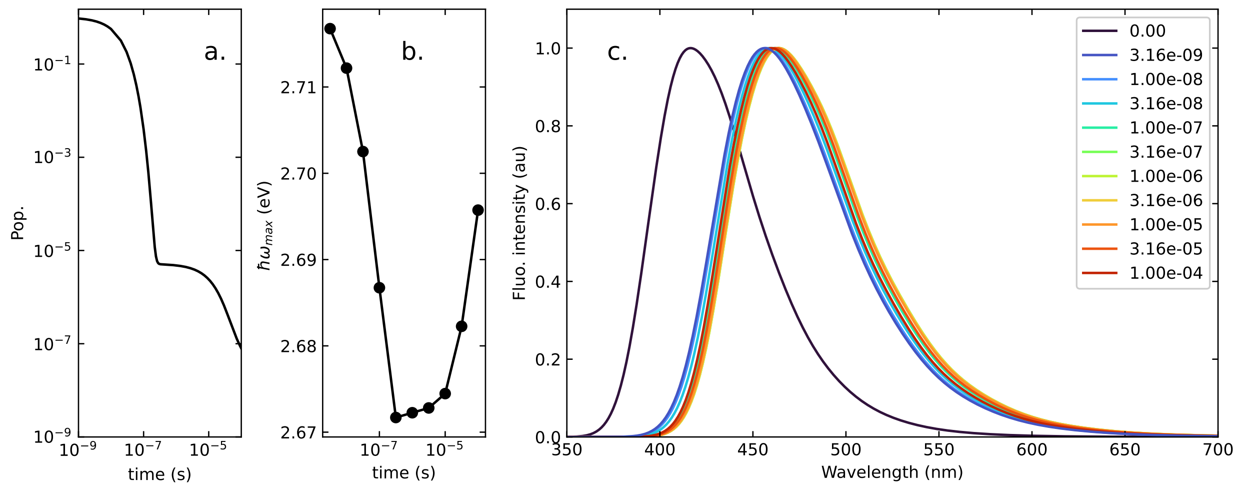

Figure S15: Simulation of the photophysics of DMAC-TRZ in Zeonex matrix, with $\hbar\omega_c = 2.0\times10^{-3}$ eV. (a) time-evolution of the singlet population (b) time-evolution of the maximum of the fluorescence spectra (c) time-resolved emission spectra. Time expressed in seconds.

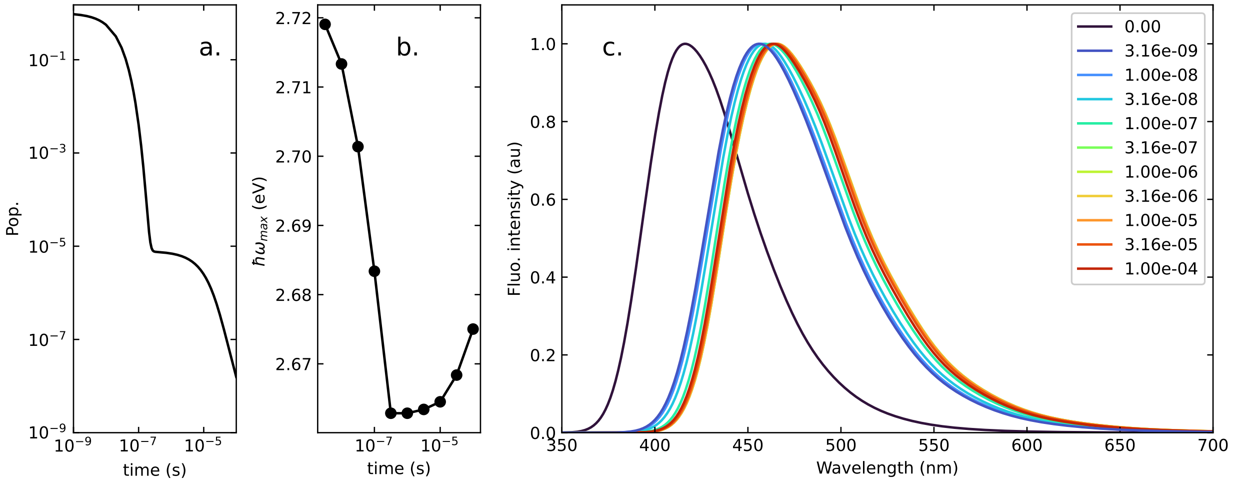

Figure S16: Simulation of the photophysics of DMAC-TRZ in Zeonex matrix, with $\hbar\omega_c = 8.0\times10^{-3}$ eV. (a) time-evolution of the singlet population (b) time-evolution of the maximum of the fluorescence spectra (c) time-resolved emission spectra. Time expressed in seconds.

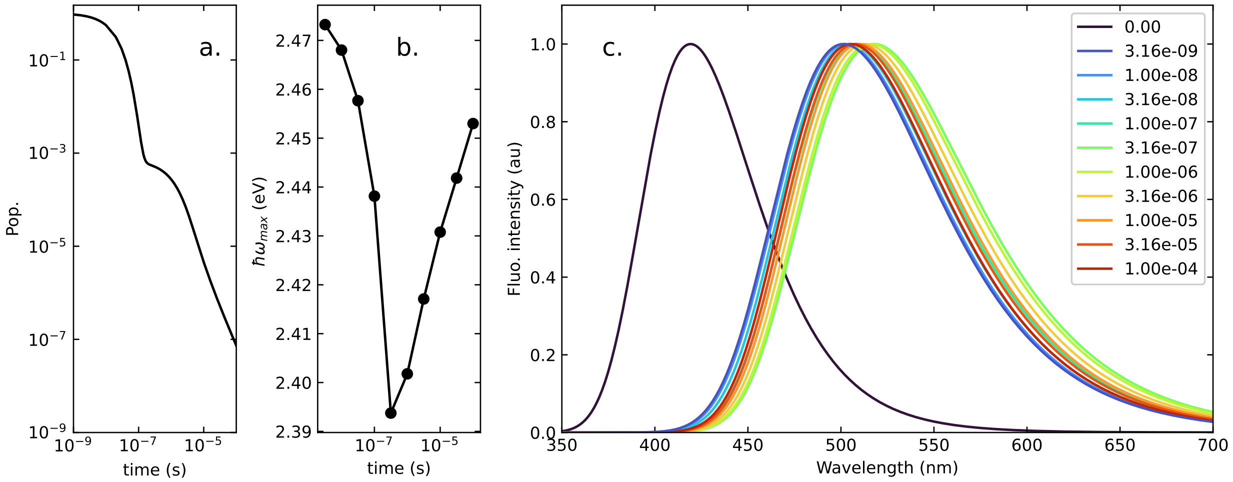

Figure S17: Simulation of the photophysics of DMAC-TRZ in DPEPO matrix, with $\hbar\omega_c = 2.0\times10^{-3}$ eV. (a) time-evolution of the singlet population (b) time-evolution of the maximum of the fluorescence spectra (c) time-resolved emission spectra. Time expressed in seconds.

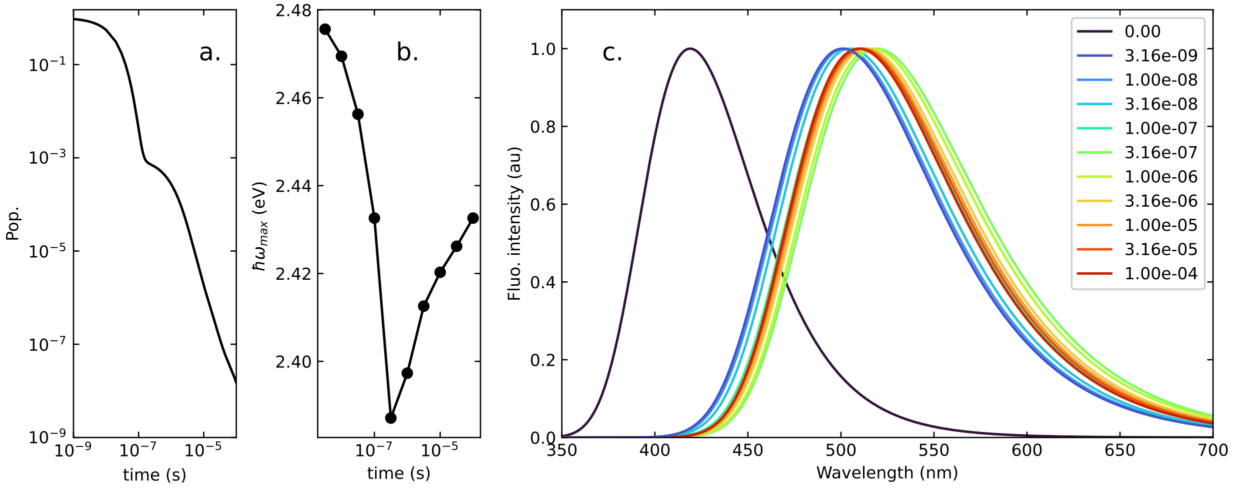

Figure S18: Simulation of the photophysics of DMAC-TRZ in DPEPO matrix, with $\hbar\omega_c = 8.0\times10^{-3}$ eV. (a) time-evolution of the singlet population (b) time-evolution of the maximum of the fluorescence spectra (c) time-resolved emission spectra. Time expressed in seconds.

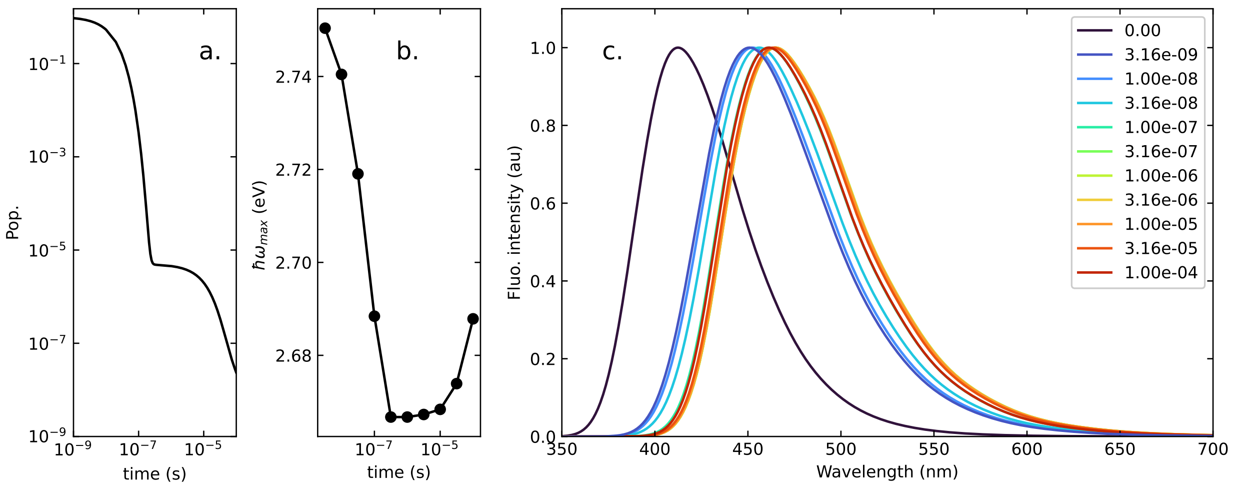

Figure S19: Same as Fig. 10, but using a broader distribution ($\sigma = 20°$) to simulate a higher degree of conformational disorder.

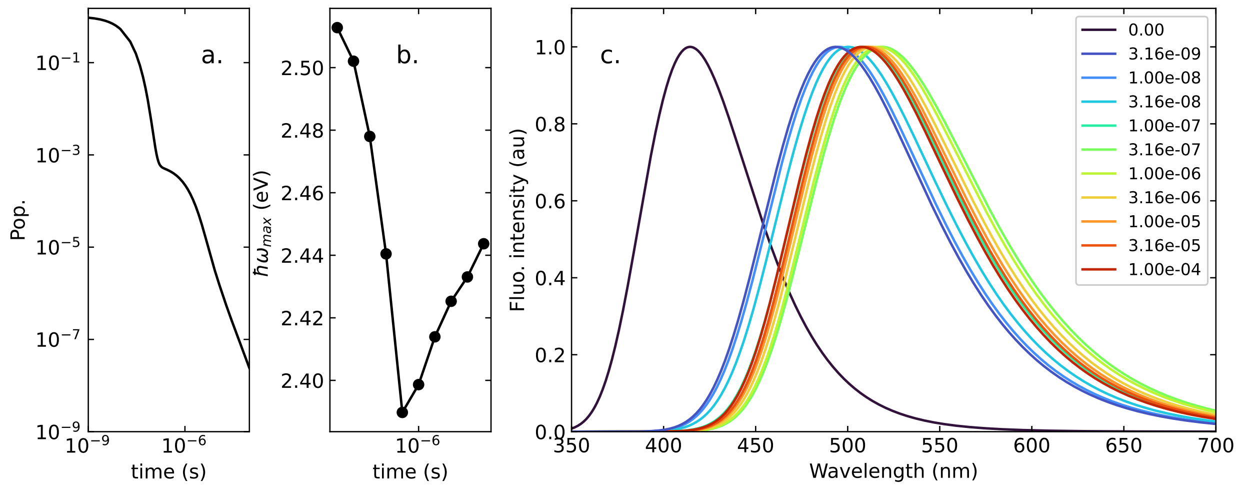

Figure S20: Same as Fig. 11, but using a broader distribution ($\sigma = 20°$) to simulate a higher degree of conformational disorder.

References

(S1) Dhali, R.; Phan Huu, D. K. A.; Bertocchi, F.; Sissa, C.; Terenziani, F.; Painelli, A. Understanding TADF: a joint experimental and theoretical study of DMAC-TRZ. *Physical Chemistry Chemical Physics* **2021**, *23*, 378–387.

(S2) Phan Huu, D. K. A.; Sissa, C.; Terenziani, F.; Painelli, A. Optical spectra of organic dyes in condensed phases: the role of the medium polarizability. *Physical Chemistry Chemical Physics* **2020**, *22*, 25483–25491.

(S3) Phan Huu, D. K. A.; Saseendran, S.; Painelli, A. Effective models for TADF: the role of the medium polarizability. *Journal of Materials Chemistry C* **2022**, 4620–4628.

(S4) Frisch, M. J.; Trucks, G. W.; Schlegel, H. B.; Scuseria, G. E.; Robb, M. A.; Cheeseman, J. R.; Scalmani, G.; Barone, V.; Petersson, G. A.; Nakatsuji, H.; Li, X.; Caricato, M.; Marenich, A. V.; Bloino, J.; Janesko, B. G.; Gomperts, R.; Mennucci, B.; Hratchian, H. P.; Ortiz, J. V.; Izmaylov, A. F.; Sonnenberg, J. L.; Williams-Young, D.; Ding, F.; Lipparini, F.; Egidi, F.; Goings, J.; Peng, B.; Petrone, A.; Henderson, T.; Ranasinghe, D.; Zakrzewski, V. G.; Gao, J.; Rega, N.; Zheng, G.; Liang, W.; Hada, M.; Ehara, M.; Toyota, K.; Fukuda, R.; Hasegawa, J.; Ishida, M.; Nakajima, T.; Honda, Y.; Kitao, O.; Nakai, H.; Vreven, T.; Throssell, K.; Montgomery, J. A., Jr.; Peralta, J. E.; Ogliaro, F.; Bearpark, M. J.; Heyd, J. J.; Brothers, E. N.; Kudin, K. N.; Staroverov, V. N.; Keith, T. A.; Kobayashi, R.; Normand, J.; Raghavachari, K.; Rendell, A. P.; Burant, J. C.; Iyengar, S. S.; Tomasi, J.; Cossi, M.; Millam, J. M.; Klene, M.; Adamo, C.; Cammi, R.; Ochterski, J. W.; Martin, R. L.; Morokuma, K.; Farkas, O.; Foresman, J. B.; Fox, D. J. Gaussian˜16 Revision B.01. 2016; Gaussian Inc. Wallingford CT.

(S5) Painelli, A. Amplification of NLO responses: vibronic and solvent effects in push–pull polyenes. *Chemical Physics* **1999**, *245*, 185–197.

(S6) Stavrou, K.; Franca, L. G.; Monkman, A. P. Photophysics of TADF Guest–Host Systems: Introducing the Idea of Hosting Potential. *ACS Applied Electronic Materials* **2020**, *2*, 2868–2881.

(S7) McHale, J. L. *Molecular Spectroscopy*, 2nd ed.; CRC Press, 2017; Chapter 6, pp 137–139.
